# Integrative genomic analysis identifies *DPP4* inhibition as a modulator of *FGF17* and *PDGFRA* downregulation and *PI3K/Akt* pathway suppression leading to apoptosis

**DOI:** 10.3389/fphar.2025.1606914

**Published:** 2025-07-22

**Authors:** Kiran Kumar Chitluri, Emerson Isaac Arnold

**Affiliations:** Bioinformatics Programming Lab, Department of Bioscience, School of Bio Sciences and Technology, Vellore Institute of Technology, Vellore, Tamilnadu, India

**Keywords:** Linagliptin, prostate cancer, apoptosis, mutation, *DPP4/CD26*, *PI3K/Akt* pathway, PC3, DU145

## Abstract

**Introduction:**

Prostate cancer (PCa) remains a significant global health challenge despite advancements in treatment strategies. There is a need to explore the molecular heterogeneity of PCa to facilitate the development of personalized treatment approaches. This study investigates the molecular heterogeneity of PCa by combining genomic and transcriptomic data using a systems biology approach.

**Methods:**

By utilising whole-genome sequencing and differentially expressed genes from “The Cancer Genome Atlas Prostate Adenocarcinoma (TCGA-PRAD)” patient samples, we identified 357 intersecting genes. From protein-protein interaction network analysis, 22 hub genes were identified as critical regulators associated with PCa prognosis and pathogenesis. Furthermore, these hub genes were subjected to functional and pathway enrichment analysis via gene ontology (GO) and the Kyoto Encyclopaedia of Genes and Genomes (KEGG).

**Results:**

Notably, the *PI3K/Akt* signalling pathway was significantly enriched with eight of these hub genes, with significant clinical relevance. Dipeptidyl Peptidase 4 (DPP4) emerged as a promising therapeutic target. Further, in vitro assays were performed to investigate and validate the molecular role of DPP4 through pharmacological inhibition using Linagliptin, a selective DPP4 inhibitor. Inhibition of DPP4 led to the induction of apoptosis, G1/S phase cell cycle arrest, and significant suppression of cell proliferation and migration in PC3 and DU145 cell lines.

**Discussion:**

These experiments revealed novel downstream regulatory effects of DPP4, demonstrating that its inhibition results in the transcriptional downregulation of *FGF17, PDGFRA, COL4A1*, and *COL9A2*, thereby contributing to the inactivation of the *PI3K/Akt* signaling pathway. Collectively, these findings highlight DPP4 as a potential therapeutic target for the treatment of PCa.

## Graphical Abstract

**Figure:**
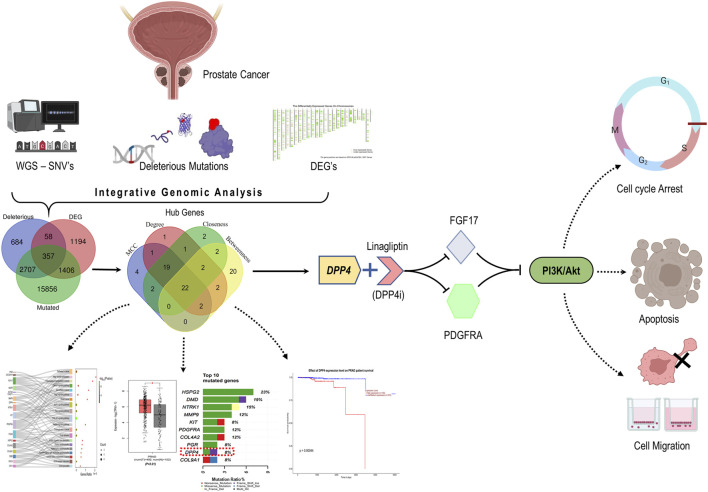
By integrative genomic analysis, DPP4 emerged as potential therapeutic target in PCa. Inhibiting DPP4 downregulates PDGFRA and FGF17 and induced cellular apoptosis, cell cycle arrest and hinders cell migration by disrupting the PI3K/Akt pathway.

## 1 Introduction

Prostate cancer (PCa) is a prevalent non-dermal heterogeneous malignancy among males that poses a significant health concern worldwide, and it remains the second leading cause of cancer-related deaths in men (Sekhoacha et al., 2022; Bhargavi et al., 2023). According to the American Cancer Society (ACS) report “Cancer Statistics 2023″, there are an estimated 288,300 cases of PCa in the United States of America alone, accounting for 29% of all cancer types among males (Siegel et al., 2023). However, it is noteworthy that while a significant number of individuals may be diagnosed with PCa, only a minority of them will exhibit clinically relevant manifestations of the disease (Silberstein et al., 2013). Treatments available for PCa consist of surgery, radiation therapy, hormone therapy, chemotherapy, and targeted therapy. However, there is a pressing requirement for improved treatments, particularly for aggressive and treatment-resistant types of the condition. The emphasis should be on identifying and diagnosing PCa accurately, particularly in less developed nations where mortality rates are higher due to limited access to screening and better treatment alternatives (Sekhoacha et al., 2022; Wasim et al., 2023; Ferroni et al., 2019).

Advancements in genomics and precision medicine hold substantial promise for identifying novel therapeutic targets in PCa. Unique genetic alterations such as mutations in tumor suppressor genes and oncogenes can promote tumor progression and therapy resistance. Notably, the androgen receptor (AR) signaling pathway, a key driver of PCa, is tightly regulated by hormonal activity, particularly testosterone and dihydrotestosterone. Dysregulation of this pathway, often through AR gene amplification, mutations, or splice variants, leads to sustained AR activation even in the absence of androgens, contributing to castration-resistant prostate cancer (CRPC) (Ma et al., 2024; He et al., 2021; Chitluri and Emerson, 2024). Additionally, genetic alterations in pathways such as PTEN/PI3K/AKT, DNA damage repair (e.g., BRCA1/2), and TMPRSS2-ERG fusions further enhance tumor aggressiveness and confer resistance to conventional hormone therapies (Waarts et al., 2022; Berger and Mardis, 2018). Integrating hormonal context with genomic analysis is thus critical, as the crosstalk between androgen signaling and oncogenic mutations shapes disease trajectory and treatment response (Li et al., 2025; Wang et al., 2008). Understanding these interactions facilitates the development of targeted therapies such as AR inhibitors, PI3K inhibitors, and PARP inhibitors that disrupt key hormonal and genetic drivers of PCa progression (Antonarakis et al., 2020; Crumbaker et al., 2017).

Similarly, gene expression profiles can provide valuable information about the biological characteristics of a tumor (Creighton, 2023). Overexpression or under-expression of certain genes can indicate the presence of specific molecular subtypes of cancer, each with distinct clinical behaviours and treatment responses. For instance, the overexpression of androgen receptor signaling pathways in metastatic PCa highlights the importance of androgen deprivation therapies (Hoang et al., 2017; Jillson et al., 2021). Nevertheless, some patients develop resistance to androgen deprivation therapy (ADT), resulting in poor prognosis and limited treatment choices (Maitland, 2021). However, as tumors evolve, they may alter their gene expression patterns to develop resistance to these therapies, necessitating the identification of new targets and the development of novel therapeutic approaches (Gatenby and Brown, 2020). We have conducted a comprehensive study to address this issue. Our study involved identifying mutated genes (MutGs), characterizing deleterious mutated genes (DMutGs) from NGS data and integrating differentially expressed genes (DEGs) retrieved from the GEPIA2 database. Additionally, we incorporated protein-protein interaction (PPI) networks, identified hub genes, predicted correlations between hub genes, and conducted Gene Ontology (GO) and Kyoto Encyclopaedia of Genes and Genomes (KEGG) pathway enrichment analyses. Furthermore, we evaluated immune cell infiltrates and performed survival analysis using large-scale DNA microarray and RNA-Seq data from GEO, TCGA, and other public databases to explore potential hub genes and biological pathways related to the occurrence, development, and prognosis of PCa. We identified 22 hub genes involved in PCa progression and metastasis, investigated them, identified potential therapeutic targets, and experimentally validated them.

## 2 Materials and methods

### 2.1 *In-silico*

#### 2.1.1 Dataset download and single nucleotide variants (SNV’s) identification from WGS

To conduct our study, we obtained the paired-end (PE) whole genome sequence (WGS) of a human adult male prostate tumor tissue in fasta format with the run SRA accession SRP250789 from the SRA database accessed on February 2023. We also obtained differentially expressed genes (DEGs) for prostate adenocarcinoma (PRAD), from the GEPIA2 database (Tang et al., 2019). These genes were selected based on specific criteria, including a log_2_FC > 1 and a q-value <0.01. To ensure the quality of the WGS reads, we subjected them to quality control and assessed the quality using FastQC. Any low-quality sequences were removed using Trimmomatic ILLUMINACLIP NexteraPE-PE for adapters, with MINLEN adopted to drop the reads below 25 bp length and SLIDINGWINDOW was invoked to remove the bases with a phred score below 20. The trimmed sequences were then aligned to the reference genome using Burrows-Wheeler aligner (BWA). Variants were detected using bcftools, and SNVs with a high-quality score of >100 were filtered.

#### 2.1.2 Determining common genes

The polyphen2 tool (Adzhubei et al., 2013) (accessed on April 2023) was used to assess the ability to predict single nucleotide variants, identify mutated genes, and predict genomes with deleterious mutations. The analysis compared deleterious mutated genes (DMutGs) to mutated genes (MutGs) and differentially expressed genes (DEGs). This investigation led to the identification of specific genes with deleterious mutations and differential expression.

#### 2.1.3 PPI networking and hub gene selection

STRING app (Szklarczyk et al., 2021) (accessed on May 2023) was used to investigate the interaction network in the common human gene set identified previously. Interaction pairs were combined with those that were experimentally verified so that an interaction scores greater than 0.4 was applied as the cut-off. Closely interacting genes were listed. We identified the top 50 hub genes based on the four topological methods: maximum clique centrality (MCC), degree, closeness, betweenness, and overlapping genes between such methods were selected.

#### 2.1.4 Enrichment analysis

In addition, the Gene Ontology (GO) analysis with the help of R “clusterProfiler” was performed to investigate further functional roles played by the overlapped hub genes and the top cluster genes. The cellular component (CC), biological process (BP), and molecular functions (MF) involved with the presented genes were determined by GO enrichment analysis. Likewise, using ShinyGO (v.0.79) (Ge et al., 2020), KEGG analysis was performed to correlate the enriched biological pathways of the hubs with edge cut-off = 0.2 and FDR *p*-value cut-off = 0.05, followed by classification of signal pathways. This enabled us to identify some putative biological processes, signaling pathways and human disease pathways that would account for PCa pathogenesis and progression.

#### 2.1.5 Kaplan-Meier overall survival (OS) analysis of hubs

To investigate the prognostic potential for identified hub genes, Kaplan-Meier curve analysis was performed to compare clinical outcomes based on the hub gene expressions associated with poor prognosis in PCa, highlighting their potential as prognostic biomarkers for identifying high-risk patients’ overall outcomes in PCa patients. In the current study, Kaplan-Meier survival analysis with hazard ratio (HR) was performed using UALCAN (Chandrashekar et al., 2017) and GEPIA2.0 databases - TCGA prostate adenocarcinoma (PRAD) cohort (accessed on August 2023). The cohort population was stratified into high- and low-expression groups based on the median hub genes expression level, ensuring an unbiased and biologically relevant comparison. (log rank p-value<0.05, p (HR) < 0.05).

#### 2.1.6 Tumor infiltration analysis

To further investigate the potential role of the identified hub genes in the tumor microenvironment, we evaluated the correlation between tumor-infiltrating immune cells across the TCGA-PRAD cohort and the expression levels of PCa hub genes using the TIMER2.0 database (accessed on September 2023) (Li et al., 2020). We used the Spearman’s test (ρ) and a p-value of <0.05 was considered statistically significant. This analysis helped us to gain insights into the potential interactions between immune cells and the identified hub genes in PCa, which may provide new avenues for therapeutic interventions.

#### 2.1.7 Estimating the expression of the hubs in correlation with DPP4

To check the expression of the other hub genes in comparison with DPP4, we used the Gene_Corr module, and the degree of correlation was established based on the “Spearman’s rho” value. These analyses helped us to evaluate the potential impact of DPP4 expression on the expression of other hub genes in PCa and identify potential therapeutic targets.

### 2.2 *In-vitro*

#### 2.2.1 Cell lines and reagents

DU145, PC3 and HEK293 were obtained from the National Center for Cell Sciences (NCCS, Pune). For cell culture, Dulbecco’s Modified Eagle Medium, High Glucose (DMEM), Fetal Bovine Serum (FBS), Antibiotic Mixture (Streptomycin and penicillin) and Cell culture grade dimethyl sulfoxide (DMSO) were purchased from HI-Media laboratories, Mumbai. RT-PCR and cDNA conversion kits were obtained from Takara, Chennai. Other plastic materials, including Transwell migration plates, were purchased from Tarson, India. Linagliptin and doxorubicin were purchased from Sigma-Aldrich, India.

#### 2.2.2 Cell maintenance and cell viability assay

DU145, PC3 and HEK293 cells were maintained in a humidified incubator set at 37°C and 5% CO2, using a complete medium including DMEM, 10% FBS, 100 IU/mL penicillin, and 100 μg/mL streptomycin. The media was changed every 2–3 days. Once the cells had achieved 80%–90% confluency, they were collected, and experimental procedures were carried out.

Cell viability was assessed by seeding cells at a density of 1 × 10^4^ per well on a 96-well plate supplemented with complete medium and incubating them overnight in a humidifier incubator. The next day, the culture medium was withdrawn, and the cells were exposed to Linagliptin at varying concentrations for 24 h. Following the specified duration, the drug was removed, and 20 µL of MTT (5 mg/mL in PBS) was added to the well. The well was then incubated in the absence of light for 4 h. After 4 h, MTT was removed, and 100 µL of DMSO was added to the wells. The intensity of the formazan crystal was then quantified at 490 nm using a Microplate Reader from Bio-Tek, United States. Three repetitions of each analysis were conducted, and the cell viability was compared to that of the untreated control group. In this work, doxorubicin served as the positive control.

#### 2.2.3 Morphological changes

PC3 and DU145 cells, exhibiting exponential growth, were collected and plated in 6-well plates at a density of 1 × 10^5^ cells per well and incubated overnight. The cells were subsequently incubated for 24 h with Linagliptin (IC50), while cells treated with Doxorubicin were the positive control. Cell morphology was analyzed using an inverted microscope (MAGNUS 10J617).

#### 2.2.4 Scratch assay

For this assay, PC3 and DU-145 cells were seeded at a density of 1 × 10^5^ cells per well in a 6-well plate and incubated for 24 h. A scratch was created on the plates utilizing a 200 μL sterile pipette tip. The plate was washed with 1XPBS to eliminate cellular debris and subsequently treated with Linagliptin (IC50) for 24 h. Control cells were kept untreated, while doxorubicin (3.5 μM) was the positive control. Images were obtained at 0^th^ and 24th-hour post-drug administration utilizing an inverted microscope (MAGNUS 10J617). Image closure was measured using ImageJ software and represented as %wound closure relative to the size at the 0^th^ hour. The percentage of wound closure is calculated as follows:

$$\%\text{ Would Closure}=\frac{\left(Original\;scratch\;width\right)-\left(new\;scratch\;width\right)}{Original\;scratch\;width}\times 100$$

#### 2.2.5 Transwell migration assay

The impact of Linagliptin on the migration of PC3 and DU145 cells was further examined using a Transwell migration experiment in 6-well Corning Transwell cell culture inserts with an 8 μm pore size. Subsequently, following treatment with Linagliptin at IC50 concentration and Doxorubicin, the cells were trypsinized and re-suspended in serum-free media. Subsequently, 200 μL (1 × 10^5^ cells) of the cell suspension was introduced into the upper compartment in serum-free media, while 1,000 μL of complete media was supplied to the lower chamber. Following 24 h of incubation, the non-migrated cells from the upper surface of the insert were eliminated using a sterile cotton swab. The migrating cells on the lower side were rinsed with PBS and fixed in 70% ethanol for 10 min. The cells were subsequently stained with 0.5% crystal violet for 20 min, followed by a PBS wash to eliminate excess stain. The quantity of migrating cells was enumerated in three distinct fields and captured using an inverted microscope (MAGNUS 10J617).

#### 2.2.6 Cell cycle analysis and determining apoptosis using annexin V-FITC

PC3 and DU145 cells (1 × 10^5^ cells/well) were treated with Linagliptin for 24 h alongside a control group without treatment. The cells were trypsinized and rinsed three times with 1X PBS before being fixed in 5 mL of 70% ice-cold ethanol for 24 h on ice. The cells were subsequently rinsed with PBS and subjected to a 10 μg/mL RNase A (Himedia, Mumbai, India) treatment at 37 °C for 30 min. The cells were stained with 50 μg/mL propidium iodide (Thermo Fisher Scientific, Bangalore, India) for 20 min in the dark. The outcomes were assessed using flow cytometry (CytoFLEX S, Beckman Coulter). Additionally, percentage of apoptosis was determined using annexin-V fluorescein isothiocyanate (FITC) and propidium iodide (PI) apoptosis detection kit (BD Biosciences) according to the manufacturer’s instructions. Percentage of apoptotic cells (annexin+/PI+) was analysed by flow cytometry (CytoFLEX S, Beckman Coulter).

#### 2.2.7 AO/EtBr staining

An EtBr and AO double staining test was conducted to evaluate Linagliptin’s impact on cellular apoptosis in PC3 and DU145 cells. The cells were cultured on coverslips and treated with Linagliptin for 24 h. Untreated control cells were maintained, whereas Doxorubicin served as the positive control. Following incubation, a staining solution comprising AO (100 mg mL^-1^) and EtBr (100 mg mL^-1^) was administered to the cells, which were then incubated in darkness for 5 min. Using a fluorescent microscope, the cells were subsequently analysed for their staining pattern (Weswox Optik-FM 3000).

#### 2.2.8 RNA isolation and RT-PCR

PC3 and DU145 cells (1 × 10^5^ cells/well) were seeded in 6-well plates and treated with Linagliptin at IC50 concentration and Doxorubicin for 24 h. RT-PCR was employed to assess the mRNA expression of target genes. RNA was extracted from cells utilizing TRI Reagent^®^ (Sigma Aldrich, Bangalore, India), and the resultant RNA was quantified using a Nanodrop 2000 (ThermoFisher Scientific, Bangalore, India). cDNA synthesis was conducted utilizing the PrimeScript™ RT reagent Kit (DSS Takara Bio India Pvt. Ltd., Bangalore, India). RT-PCR was done using SYBR^®^ Premix Ex Taq™ (DSS Takara Bio India Pvt. Ltd., Bangalore, India). Each real-time PCR utilized 100 ng of RNA. The quantitative RT-PCR was conducted utilizing the Light Cycler 2.0 (Applied Biosystems^®^ StepOne RT-PCR equipment, Bangalore, India). A melting curve study was conducted post-amplification utilizing LightCycler software (A.B. Biosystems, India). Beta-actin served as an internal standard, and the results were presented as fold change over relative control. Primer list is added in Table 1.

**TABLE 1:** Primers list.

Gene Name	Forward Primer (5′-3′)	Reverse Primer (5′-3′)
*PIK3CA*	GGTTGTCTGTCAATCGGTGACTGT	GAACTGCAGTGCACCTTTCAAGC
*AKT1*	TTCTGCAGCTATGCGCAATGTG	TGGCCAGCATACCATAGTGAGGTT
*PTEN*	*GGTTGCCACAAAGTGCCTCGTTTA*	*CAGGTAGAAGGCAACTCTGCCAAA*
*FGF17*	GTGTTCACGGAGATCGTGCTG	GAACTGCTTCTGCTTCTCGGC
*PDGFRA*	GACTTTCGCCAAAGTGGAGGAG	AGCCACCGTGAGTTCAGAACGC
*COL4A1*	TGTTGACGGCTTACCTGGAGAC	GGTAGACCAACTCCAGGCTCTC
*COL9A2*	TGGAGTGGAAGGACCAAGAGGA	GTGCTGATCTGTCGGTGCTCTA
*Beta-Actin*	AGTCCTGTGGCATCCACGAA	GATCCACACGGAGTACTTGC

#### 2.2.9 DPP4 activity assay

DPP4 activity assay kit (MAK088) was obtained from Sigma-Aldrich, Bangalore, Karnataka, India. Cells were treated as previously described. DPP4 activity was performed based on the kit protocol.

#### 2.2.10 Statistical analysis

GraphPad Prism 8.0 was used to do statistical analysis. The results are expressed as mean ± SD of three independent experiments. One-way ANOVA with Bonferroni’s multiple comparisons was used to evaluate differences. P < 0.05 is considered significant.

#### 2.2.11 Western blot analysis

Western blot analysis was performed using PC3 cells and DU145 cells, both treated with varying concentrations of Linagliptin (0, 20, and 40 µM for PC3 and 0, 20, and 60 µM for DU145) for 24 h. After treatment, total protein was extracted by resuspending the cells and lysing them for 1 h at 4°C in RIPA (radioimmunoprecipitation assay) buffer (Cat. No. R0278) with protease and phosphatase inhibitor cocktail. The cell lysates were then centrifuged at 12,000 rpm for 30 min at 4°C, and the Bradford assay was used to determine the protein concentration. An equal volume of 20 µg protein lysate from each sample was subjected to SDS-PAGE and subsequently transferred onto a nitrocellulose membrane (Bio-Rad, cat. no. 1620112). The membrane was blocked with 5% skimmed non-fat dry milk and incubated overnight at 4°C with various antibodies: anti-FGF17 (1:2000), anti-PI3K p-85α (1:1,000), anti-AKT (1:2000), anti-p-AKT (1:2000), anti-GAPDH (1:2000), and anti-Actin (1:1,000). Following this, the membranes were rinsed three times with TBS (Tris buffered saline), each for 10 minutes, and then incubated with HRP-conjugated secondary anti-mouse or anti-rabbit antibodies (diluted to 1:5000) for 1 h at room temperature. They were washed three more times with TBST (Tris buffered saline containing Tween-20, pH 7.5). Finally, the immunoblots were visualized using ECL substrate (Clarity™ western ECL substrate Cat# 170-5061) under the ChemiDoc MP Imaging System (Bio-Rad). All experiments were conducted in triplicates.

## 3 Results

### 3.1 *In-silico*

#### 3.1.1 Variant calling

To obtain the full set of SNV data, we retrieved the whole genome sequence of a human adult male prostate tumor sample bearing the SRP250789 from ENA at https://www.ebi.ac.uk/ena/browser/view/SRP250789. We initially performed variant calling to identify the SNVs and the reads were aligned with the reference sequence. A total of reads with a quality score of <100 were retained and used for further analysis. These SNVs were mapped to the genes using BioMart (Kinsella et al., 2011), and we identified 20,326 mutated genes (MutG).

#### 3.1.2 Characterising the SNVs identified

**FIGURE 1:**
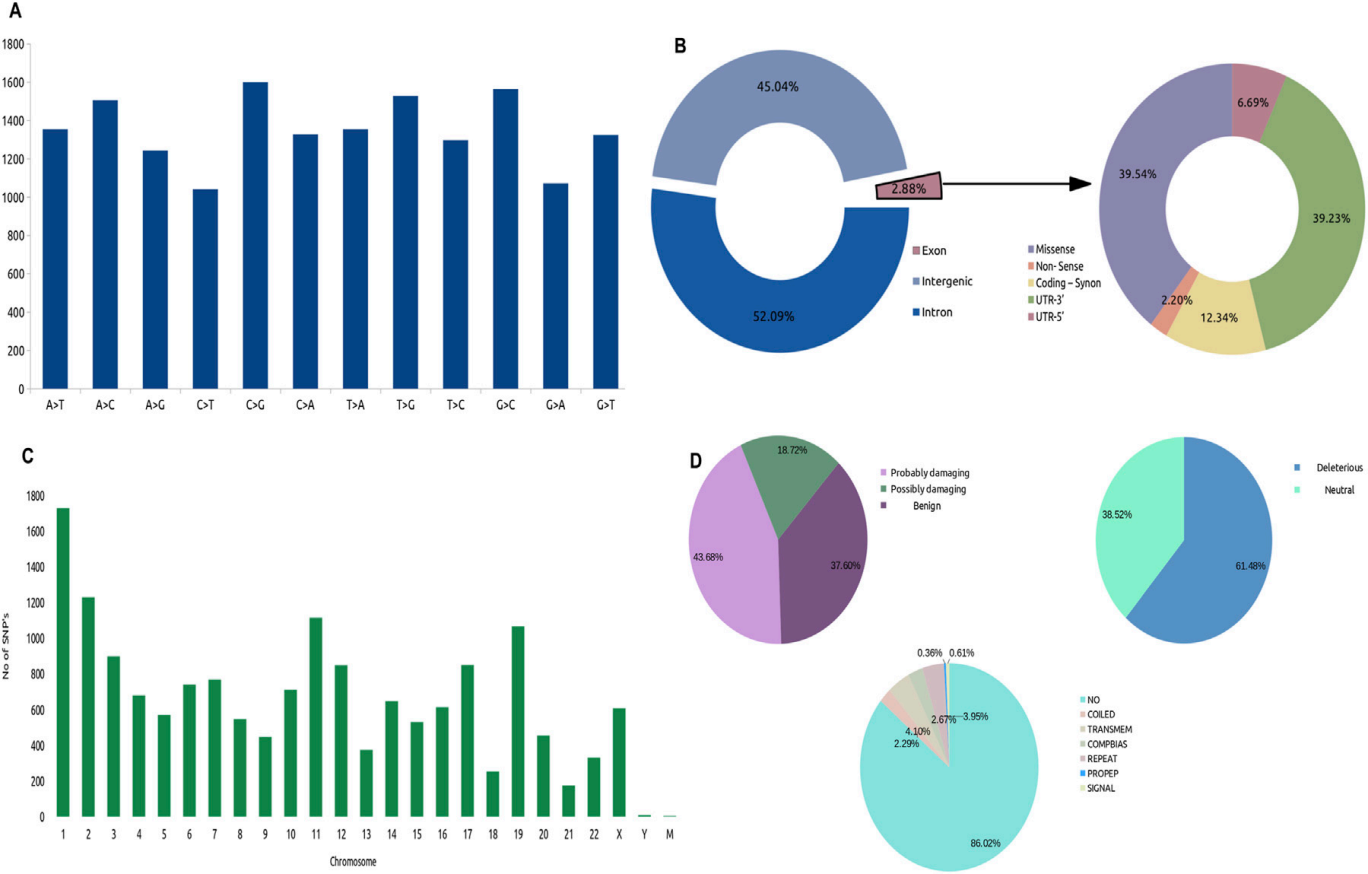
Distribution of SNV’s in the Whole Genome Sequence (WGS), **(A)** Identified types of SNV’s variations **(B)** Landscape of Missense mutations across Chromosomes, **(C)** Fractions of mutations across whole genome, **(D)** Types of exonic mutations: Characterization of Missense mutations, Left: Percentage of mutations that are probably damaging, possibly damaging and benign; Right: Percentage of mutations that are deleterious and neutral; Bottom: Location of mutations.

To further investigate the SNVs identified in the whole genome sequence, we annotated them using PolyPhen2 based on chromosomal position and discarded multi-mutations SNVs. Only 2.88% of single-nucleotide variants (SNVs) were found in exons, which are dispersed in CDS and non-sense UTRs while a large portion was located in introns (52.09%) and intergenic regions (45.05%). These areas may contain mutations in regulatory elements, such as enhancers, silencers, and non-coding RNAs. Our main goal is to identify genes that change the protein sequence through missense, nonsense, or frameshift mutations, which can disrupt protein function and lead to cancer. Although mutations in non-coding regions (introns) can affect gene expression and may contribute to cancer, we further integrated exonic mutations with the differentially expressed genes (DEGs) from the TCGA-PRAD dataset. We identified that 39.54% (16,202) of the exonic mutations were missense mutations (Figure 1B), with C>G and G>C transitions being the most prevalent (Figure 1A). These missense mutations were annotated to 16,055 genes with at least one missense mutation. They were distributed across the chromosomes, with chromosome one having the most missense SNVs (Figure 1C). Out of these genes, we identified that 18.72%, 37.60%, and 46.68% were possibly damaging, benign, and probably damaging at the functional level, respectively. We observed that 61.48% of the mutated genes were affected by deleterious mutations that impaired protein functionality (Figure 1D). In this analysis, we focused on 3,806 unique genes that had mutations that deleteriously affected protein function (DMutGs), allowing us to gain insights into the potential genetic causes of PCa and identify potential therapeutic targets.

#### 3.1.3 Common genes identification

**FIGURE 2:**
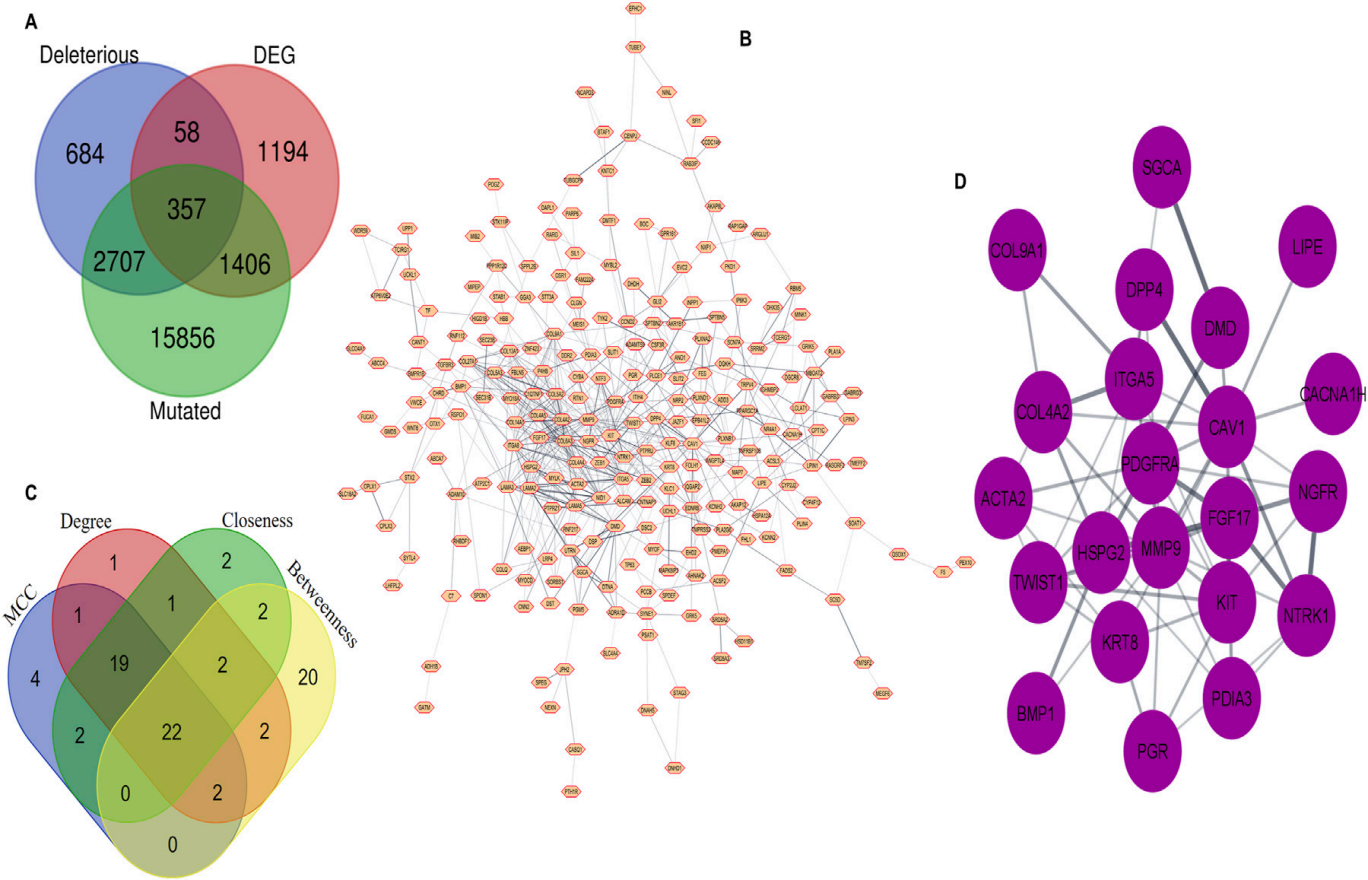
Integration of WGS and Trascriptomic data through PPI network analysis, **(A)** Common genes among Deleterious mutated genes, Differentially Expressed Genes and Mutated genes **(B)** Protein–Protein interaction of 357 common genes. **(C)** Hub genes identification by overlapping between the top 50 gene ranks based on four topological algorithms. **(D)** 22 hub genes.

To identify the most optimal key candidate gene with potential therapeutic implications, we obtained a list of 3,015 (PRAD) differentially expressed genes (DEGs) with tissue code: PRAD of GEPIA2 database using the limma differential method, with log_2_FC cut off >1 and q-value cut off <0.01. We combined the MutGs, DMutGs and DEGs and identified 357 genes (Supplementary Table S1) present in all three sets, as shown in Figure 2A.

#### 3.1.4 PPI network construction and hub selection

We utilized the Cytoscape STRING (Shannon et al., 2003) plugin to construct a PPI network for the list of 357 genes, with an interaction score set to 0.4. The network includes proteins that physically interact with at least one other member consisting of 239 nodes and 575 edges, as shown in Figure 2B. We then used cytoHubba to rank the top 50 genes based on four topological algorithms scoring values Supplementary Table S2 (i.e., MCC, degree, closeness, and betweenness) to identify the hub genes in the PPI network. We found that 22 hub genes overlapped within the four topological algorithms. This gene set was used for further analysis, as shown in Figure 2C. The identified hub genes are Collagen type IV alpha two chain *(COL4A2),* Dystrophin *(DMD)*, Receptor tyrosine kinase *(KIT)*, Sarcoglycan alpha *(SGCA)*, Neurotrophic receptor tyrosine kinase 1 *(NTRK1)*, Actin alpha 2 smooth muscle *(ACTA2)*, Caveolin-1 *(CAV1)*, Lipase E *(LIPE)*, Integrin Subunit Alpha 5 *(ITGA5)*, Calcium channel, voltage-dependent, T type alpha 1H subunit *(CACNA1H)*, Twist-related protein 1 *(TWIST1),* Progesterone receptor gene *(PGR)*, Fibroblast growth factor 17 *(FGF17)*, Nerve Growth Factor Receptor *(NGFR)*, Bone morphogenetic protein 1 *(BMP1)*, Keratin 8 *(KRT8)*, Heparansulfate proteoglycan 2 *(HSPG2)*, Platelet-derived growth factor receptor alpha *(PDGFRA),* Collagen type IX alpha one chain *(COL9A1),* Dipeptidyl peptidase 4 *(DPP4)*, Matrix metallopeptidase 9 *(MMP9),* and Protein disulfideisomerase family A, member 3 *(PDIA3)* Figure 2D.

#### 3.1.5 Enrichment analysis

**FIGURE 3:**
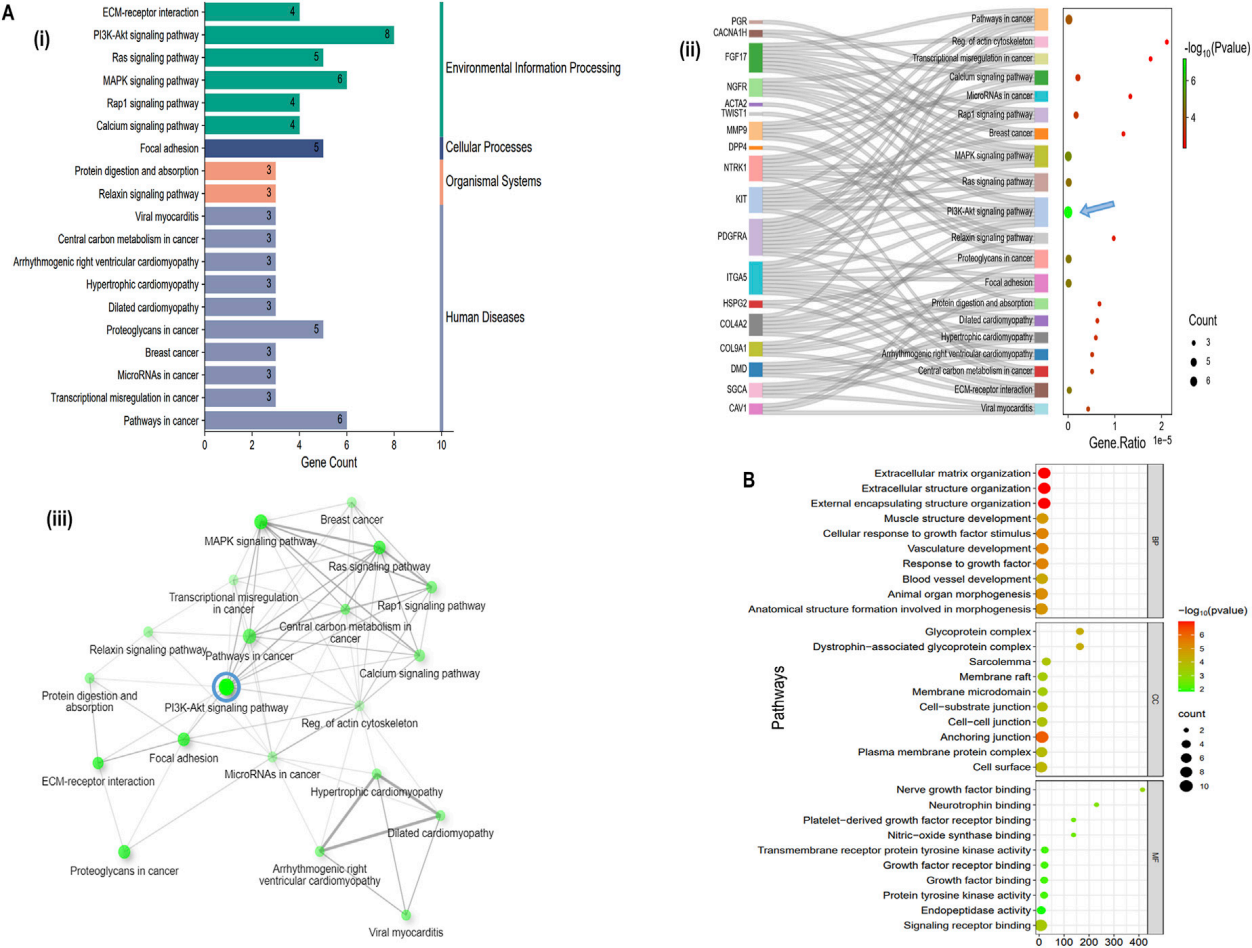
Functional enrichment of Hub genes, **(A (i)** Classification summary diagram of the top 30 KEGG pathways. **A (ii)** KEGG pathway enrichment for the identified 22 hub genes, **A (iii)** KEGG pathways network interaction (Edge cut-off = 0.2 and FDR p-value cut-off = 0.05), **(B)** Gene Ontology (GO) enrichment analysis of 22 hub genes.

The top 10 gene sets were enriched for biological process (BP), molecular function (MF), cellular component (CC) (log_10_FDR p-value <0.05), and KEGG pathways were identified using the online tool ShinyGO and illustrated the signal pathway classification. The 22 hubs were found to be enriched for various processes, including extracellular matrix organization, extracellular structure organization, external encapsulating structure organization, muscle structure development, cellular response to growth factor stimulus, vasculature development, response to growth factor, blood vessel development, animal organ morphogenesis and anatomical structure formation involved in morphogenesis (BP). (CC) analysis revealed enrichment for anchoring junction, cell-cell junction, cell-substrate junction, membrane micro-domain, membrane raft, sarcolemma, dystrophin-associated glycoprotein complex, plasma membrane protein complex, cell surface and glycoprotein complex. (MF) analysis showed enrichment for signaling receptor binding, endopeptidase activity, protein tyrosine kinase activity, growth factor binding, growth factor receptor binding, transmembrane receptor protein tyrosine kinase activity, nitric-oxide synthase binding, Platelet-derived growth factor receptor binding, neurotrophin binding and nerve growth factor binding (Figure 3B). KEGG pathway analysis revealed that hubs gene enrichment majorly in *PI3K/Akt* pathway, pathways in cancer, focal adhesion, protein digestion and absorption, and ECM-receptor interaction (log_10_FDR p-value <0.05) (Figure 3Aii). Interaction between the enriched KEGG pathways (Edge cut-off = 0.2 and FDR p-value cut-off <0.05) are depicted in Figure 3Aiii and predominantly involves human disease pathways such as the cancer pathways and proteoglycans in cancer (Figure 3Ai).

#### 3.1.6 Overall Survival analysis of hub genes

**FIGURE 4:**
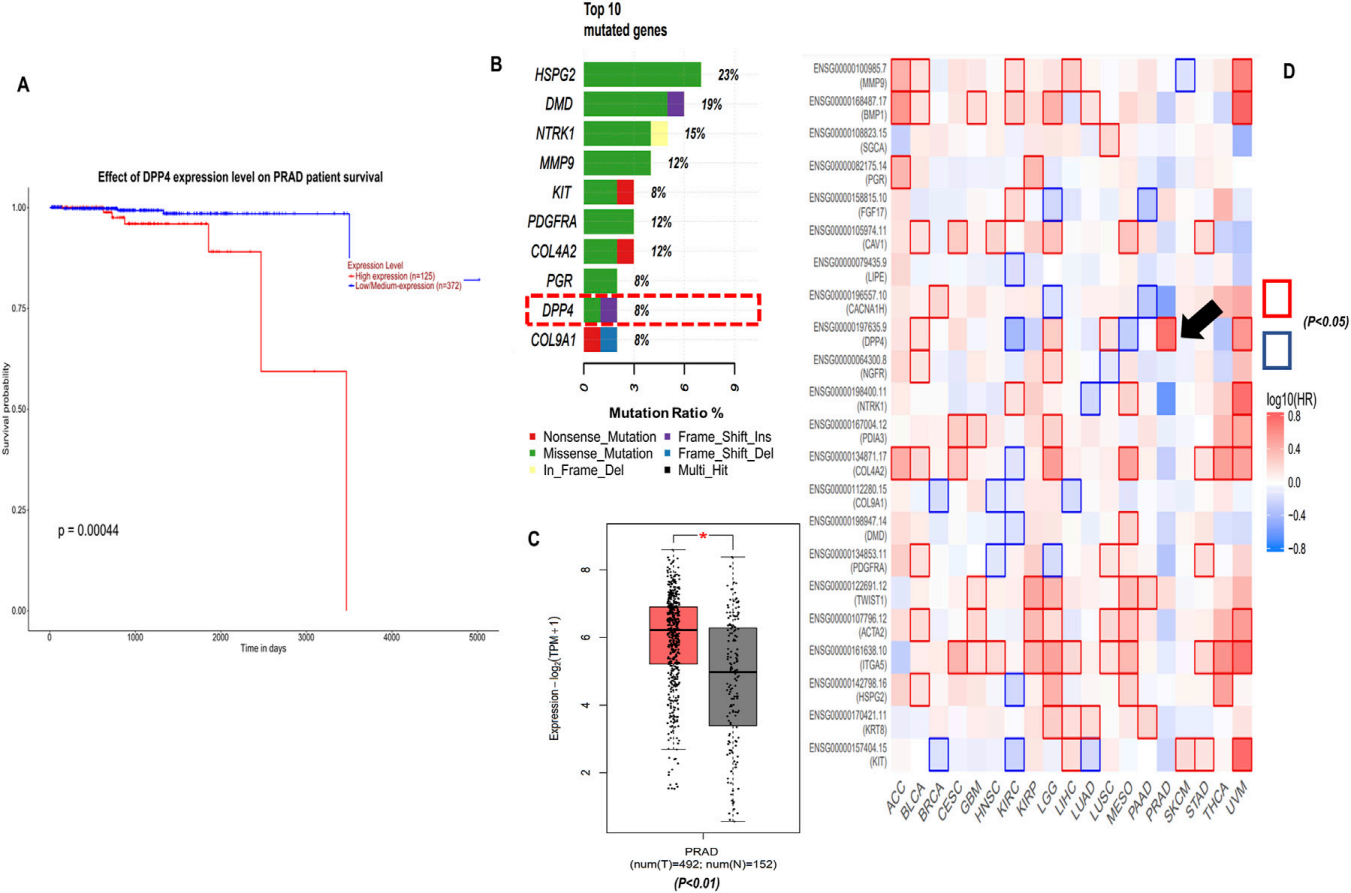
Clinical significance of DPP4, **(A)** Kaplan-Meier (KM) overall survival analysis of DPP4 using UALCAN database TCGA-PRAD cohort (log-rank p-value <0.001). **(B)** Top 10 mutated genes among 22 hub genes, **(C)** DPP4 gene expression TCGA-PRAD-GEPIA2 database, **(D)** Survival map of hazard ratio (HR) for the hub genes across cancers types with at least one significant HR (log_10_ (HR) p < 0.05).

To explore the prognostic value and clinical outcome of the hub genes, we used UALCAN (database) – to perform Kaplan-Meier overall survival analysis using TCGA–PRAD cohort, wherein the population was stratified into high–and low–expression groups based on the median hub genes expression level, ensuring an unbiased and biologically relevant comparison. The findings suggest that only DPP4 was significantly upregulated in PCa and affected patients’ overall survival (log-rank p-value <0.001) while the other 21 hub genes did not show any significant association Figures 4A,C (Supplementary Figure S3) summarises these findings. Further heat map of survival hazard ratio log_10_ (HR) p < 0.05 of hub genes depicted using GEPIA2.0 found that only DPP4 was significant, depicting prognostic implication in PRAD (Figure 4D). It was also found that DPP4 was among the top 10 highly mutated hub genes as depicted in Figure 4B.

#### 3.1.7 Tumor infiltration analysis

**FIGURE 5:**
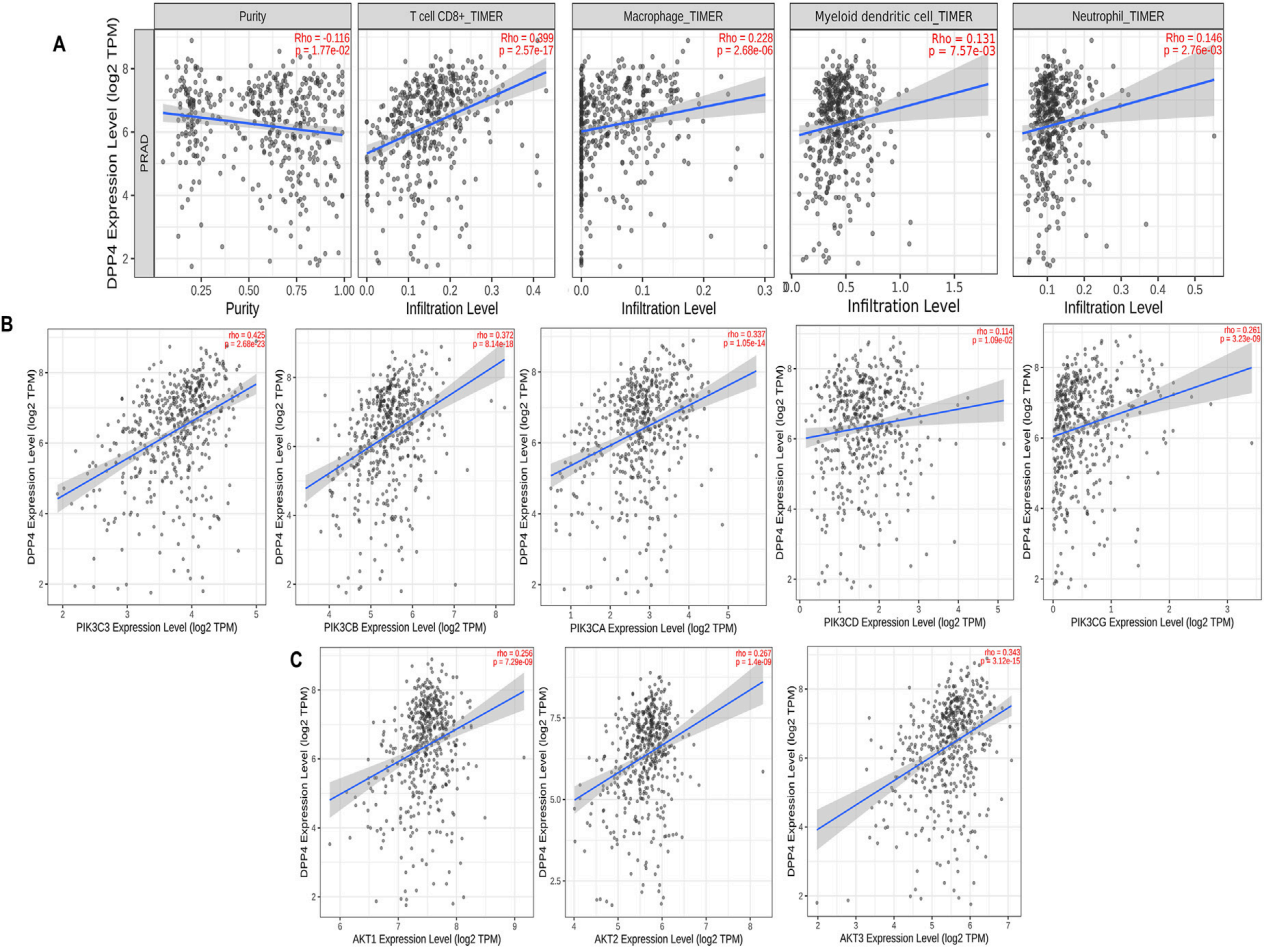
Tumor infiltration analysis of DPP4 using TIMER2.0 database, **(A)** Scatter plots that show significant positive correlations of DPP4 expression with the infiltrating levels of T cell CD8^+^, macrophage, dendritic cell, and neutrophils. **(B)** Scatter plots that showing DPP4 expression significant positive correlations with class IA PIK3 expressions. **(C)** Scatter plots that showing DPP4 expression significant positive correlations with AKT. expressions. The Spearman’s correlation value and the estimated statistical significance are displayed as the legends for each scatter plot (p-value< 0.05 (positive); p-value >0.05 (negative)).

The tumor microenvironment (TME) is essential for cancer progression, supporting tumor growth and invasion. Our survival analysis showed that higher DPP4 levels correlate with worse outcomes. We analyzed tumor infiltration to determine if DPP4 promotes or suppresses tumor growth and affects the TME through immune cell activity, suggesting DPP4 as a potential therapeutic target in PCa. Using the TIMER2.0 database, we explored the relationship between DPP4 expression and tumor-infiltrating immune cells in the prostate adenocarcinoma cohort (PRAD). The results revealed a significant positive correlation between DPP4 expression and the levels of various immune cells: CD8^+^ T cells (rho = 0.399, p-value = 2.57e-17), myeloid-derived dendritic cells (rho = 0.131, p-value = 7.57e-03), macrophages (rho = 0.228, p-value = 2.86e-04), and neutrophils (rho = 0.146, p-value = 2.76e-03), as shown in Figure 5A. Additionally, we performed a correlation analysis between DPP4 and the expression of class IA PIK3/Akt pathway components (PIK3C3, PIK3CA, PIK3CB, PIK3CD, PIK3CG) and the AKT family (AKT1, AKT2, AKT3), as revealed by pathway enrichment analysis. This analysis demonstrated a positive correlation with PIK3 (rho = 0.423, p-value = 2.68e-23), (rho = 0.337, p-value = 1.05e-14), (rho = 0.372, p-value = 8.14e-18), (rho = 0.114, p-value = 1.09e-02), and (rho = 0.261, p-value = 3.23e-09). Further, we observed positive correlations for AKT (rho = 0.256, p-value = 7.29e-09), (rho = 0.267, p-value = 1.4e-09), and (rho = 0.343, p-value = 3.12e-15), as shown in Figures 5B,C.

### 3.2 *In-vitro*

#### 3.2.1 *In-vitro* cytotoxicity and DPP4 activity

**FIGURE 6:**
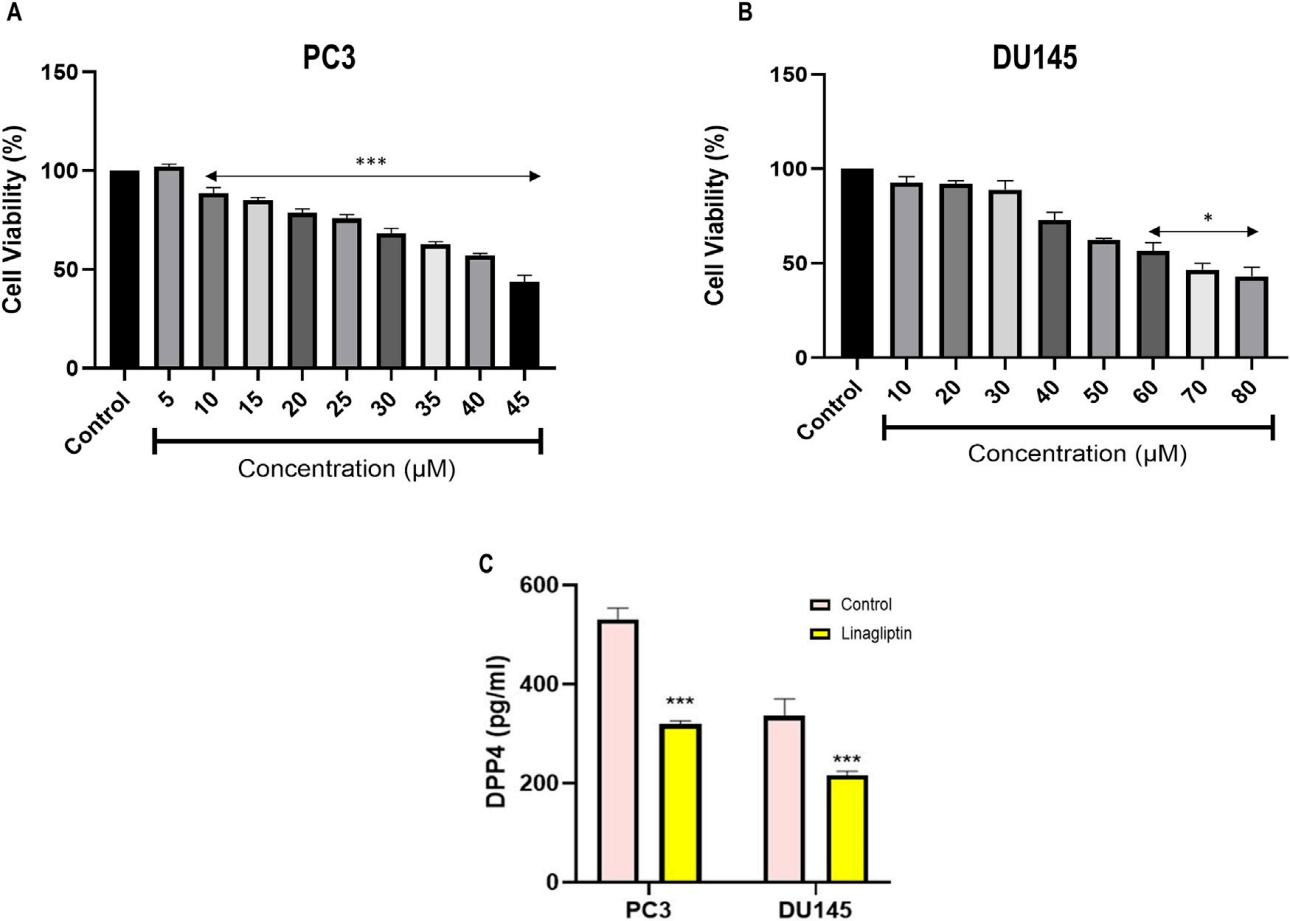
Cell cytotoxicity evaluation of Linagliptin against different cell lines, **(A)** PC3 and **(B)** DU145 prostate cancer cells. **(C)** DPP4 enzyme activity based in the Linagliptin treatment with respective IC50 values in PC3 and DU145. The results are expressed in mean ± SD of three independent experiments. ***P < 0.001 vs. Control, *P < 0.05 vs. Control.

Typically, minimal cytotoxicity to noncancerous cells and targeted elimination of malignant cells are essential criteria for an optimal antineoplastic agent. Quantitatively assessing mitochondrial integrity with a 3-(4, 5-dimethylthiazol-2-yl)-2, 5-diphenyltetrazolium bromide (MTT) assay is an excellent method for detecting the cellular proliferation index in various cytotoxic compounds. Linagliptin treatment for 24 h in PC3 and DU145 effectively reduced cell viability in a dose-dependent manner with an IC50 value of 40 μM in PC3 (Figure 6A) and 60 μM in DU145 cells (Figure 6B) with less to moderate toxicity in normal human embryonic kidney cells. Further, we assess the morphological change induced by Linagliptin using an inverted microscope. As shown in Supplementary Figure S1, Linagliptin at IC50 concentration significantly altered cell morphology. Specifically, cells appeared to have a rounded shape with subsequent detachment from the surface. However, in normal untreated control, cells showed an intact morphology without cellular death, which suggests that Linagliptin at this particular concentration can induce cellular death and subsequently change the morphology of PCa cells. Further, we validated the effect of Linagliptin on cellular DPP4 activity in its IC50 values in PC3 and DU145. As depicted in Figure 6C, both the PCa cells exhibited a higher DPP4 activity; however, after Linagliptin treatment, the activity had reduced significantly as hypothesized (P < 0.001). Further studies have been performed to confirm Linagliptin’s molecular mechanism of action to induce cellular death in cancer cells.

#### 3.2.2 Linagliptin decreased *PI3K/Akt* gene expression in PCa cells

**FIGURE 7:**
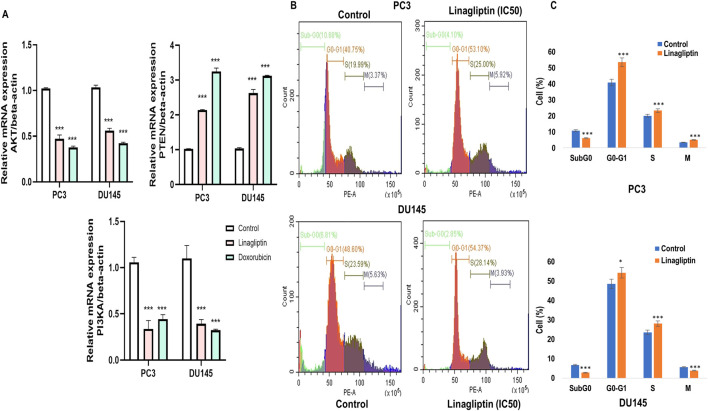
RT-PCR gene expression and cell cycle arrest. **(A)** *PI3K/AKT/PTEN* expression in PC3 and DU145 cell lines **(B)** DPP4 inhibition with Linagliptin induces of Cell cycle arrest in PC3 and DU145 cell lines. The results are expressed in mean ± SD of three independent experiments. ***P < 0.001 vs. Control, *P < 0.05 Vs. Control. **(C)** Bar graph representing the quantitative of the different cell cycle stages in both PC3 and DU145.

To further confirm our hypothesis, we checked the expression level of PI3K and AKT along with *PTEN* in Linagliptin-treated PC3 and DU145 cells. Linagliptin treatment at IC50 concentration significantly reduced the expression of *PI3KA* and *AKT* expression in PC3 and DU145 cells. We have also checked the PI3K negative regulator *PTEN* status, which acts as a tumour suppressor, and various studies have shown that the expression of PTEN influences the activation of the *PI3K/Akt* pathway. Our study revealed that the expression of *PTEN* after Linagliptin treatment significantly increased in both the cell lines, similar to Doxorubicin, suggesting *PI3K/Akt* inactivation, inhibition of cell proliferation and cell survival (Figure 7A).

#### 3.2.3 Linagliptin induces cell cycle arrest in both PC3 and DU145 PCa

Due to inadequate DNA repair mechanisms, the administration of chemotherapeutic agents typically halts the cell cycle and induces apoptosis in cancer cells. To ascertain if Linagliptin influences any cell cycle phase of PC3 and DU145 cells, we monitored the cell cycle in four phases: Sub G0, G0/G1, S, and G2/M in the treated population using flow cytometry following propidium iodide (PI) staining.

Linagliptin influences cell cycle progression in PC3 and DU145 (Figures 7B,C). 24 h of Linagliptin treatment significantly increased cellular arrest in the G0/G1 phase by 13% and S phase by 4% for PC3 cells; whereas in DU145, Linagliptin induced cell cycle arrest in the G0-G0/G1 phase by 6% followed by the S phase by 5% compared to the control group. Further the doses of Linagliptin were given to PC3 and DU145 cells for 24 h. Apoptotic cell death was determined by flow cytometry using the annexin V-FITC/PI kit. Doses of Linagliptin-40 µM - PC3, 60 µM - DU145, had significantly increase percentages of apoptotic cells in PC3 and DU145 respectively (Figure 12).

#### 3.2.4 Cellular migration after Linagliptin treatment

**FIGURE 8:**
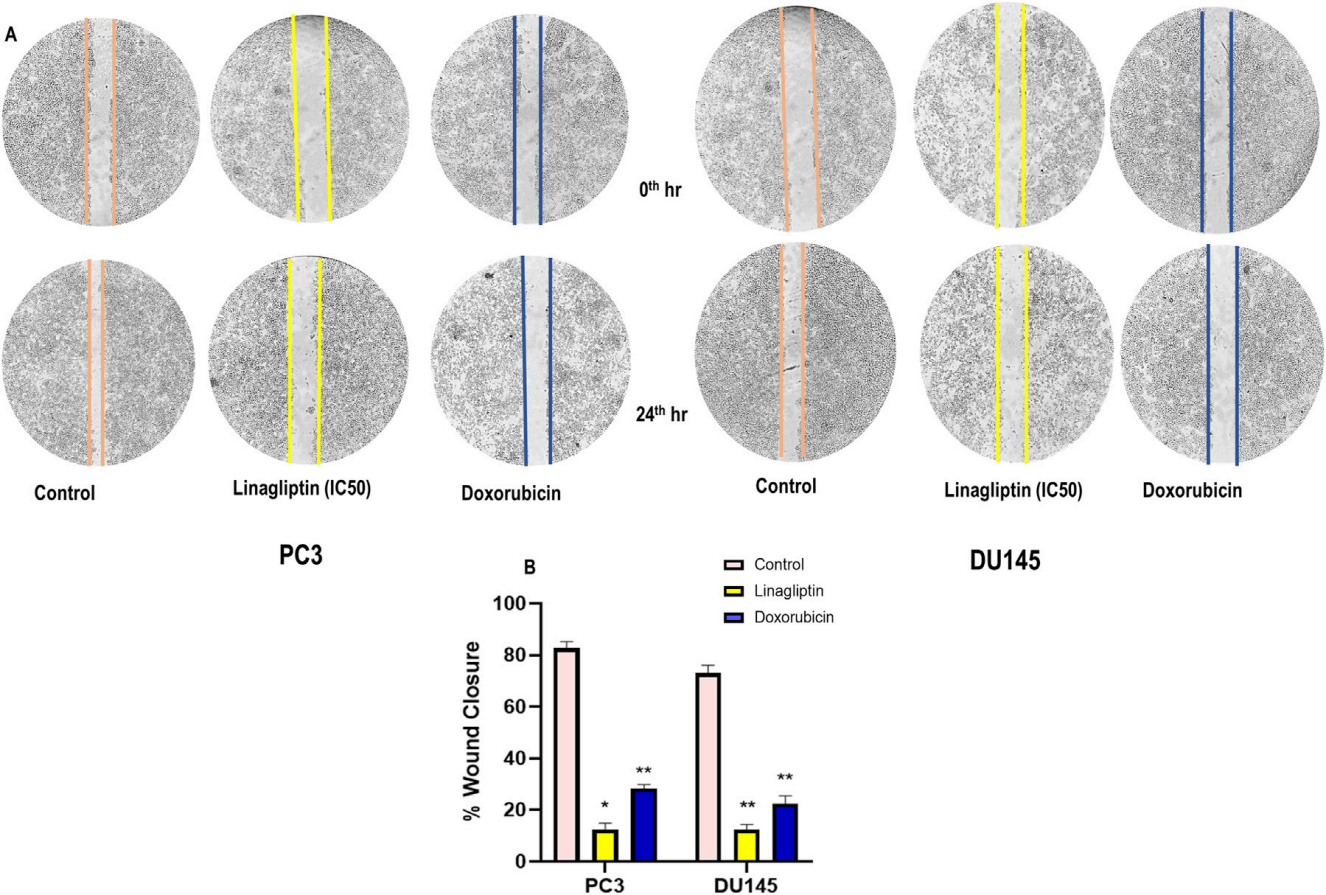
**(A)** Wound healing assay on prostate cancer cells (PC3 and DU145) treated with Linagliptin and Doxorubicin (Positive Control), **(B)** Bar graph representing the quantification of the wound healing. Results were presented graphically and the results are expressed in mean ± SD of three independent experiments. ***P < 0.001 vs. Control, **P < 0.01 Vs. Control, *P < 0.05 Vs. Control.

**FIGURE 9:**
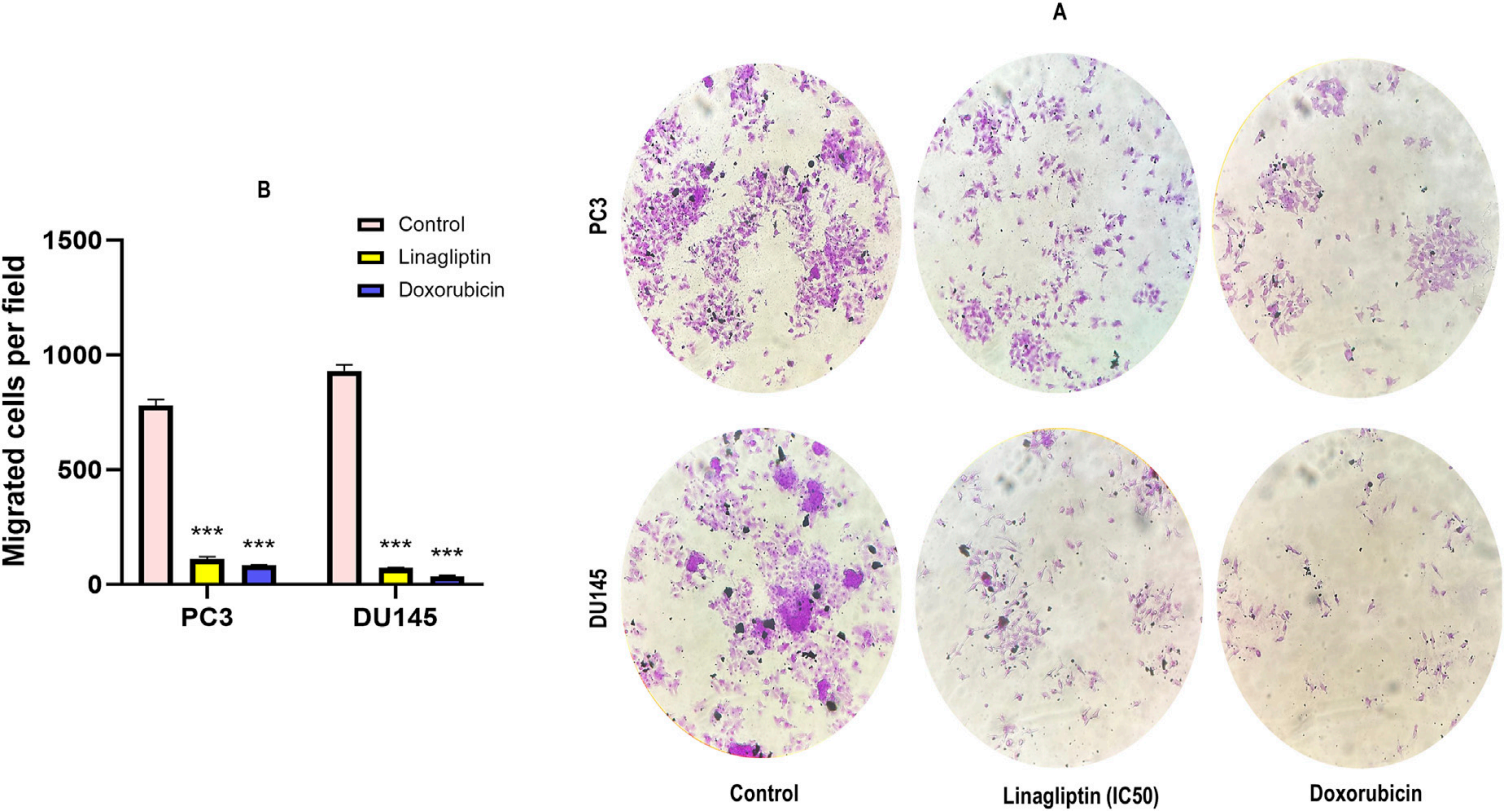
Transwell cell migration assay. **(A)** Representative photographs of migratory PC3 and DU145 treated with Linagliptin and Doxorubicin. **(B)** The results are expressed in mean ± SD of three independent experiments. ***P < 0.001 vs. Control.

Metastasis entails disseminating cancer cells from the primary tumor to secondary locations, facilitated by cellular migration and invasion within circulatory systems and the tissue matrix. We examined the impact of Linagliptin on the migratory capacity of PC3 and DU145 cells *in vitro* by a wound healing migration test. Transwell migration assay illustrates that Linagliptin impeded the migratory capacity of PC3 and DU145 cells, as indicated by the *in vitro* cytotoxicity reduction in the rate of wound closure relative to the control cells Figures 8A,B. The transwell migration assay revealed a significant reduction in the number of migrated cells in those treated with Linagliptin compared to the untreated control. This suggests that Linagliptin can impede the cellular migration of PC3 and DU145 cells *in vitro*, hence altering the metastatic potential of this aggressive prostate cancer cell type Figures 9A,B.

#### 3.2.5 DPP4 inhibition regulates hub genes in PC3 and DU145 cell line

**FIGURE 10:**
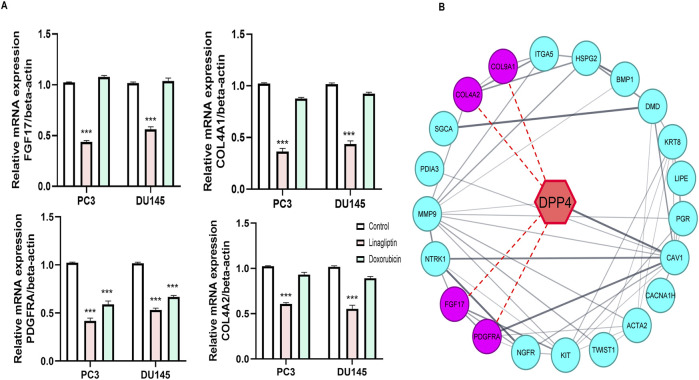
Effect of Linagliptin on Hub genes **(A)** Expression of *FGF17, COL4A1, PDGFRA, COL9A1* **(B)** Red dotted lines indicate possible interaction of DPP4 with *FGF17, COL4A1, PDGFRA, COL9A1*. The results are expressed in mean ± SD of three independent experiments. ***P < 0.001 vs. Control.

Our study further confirms the status of hub genes, which regulate the *PI3K/Akt* pathway. Interestingly, Linagliptin regulates not all but most of the hub genes at the transcription level. Treatment with Linagliptin (DPP4i) for 24 h decreased the expression of *FGF17* and *PDGFRA* in transcription level. *FGF17* and PDGF are two major growth factors that regulate the *PI3K/Akt* pathway and are associated with its activation during cancer progression. A decrease in their expression inevitably decreased the activation of the *PI3K/Akt* pathway. Moreover, Linagliptin downregulated the expression of *COL4A1* and *COL4A2*, which encodes collagenase in the tissue (Figure 10A). However, Linagliptin was found to have no role in KIT expression, which encodes tyrosine kinase receptors. The possible interaction of DPP4 with *PDGF, FGF17, COL4A1* and *COL9A2* is depicted in Figure 10B.

#### 3.2.6 Linagliptin induces apoptosis in PCa

**FIGURE 11:**
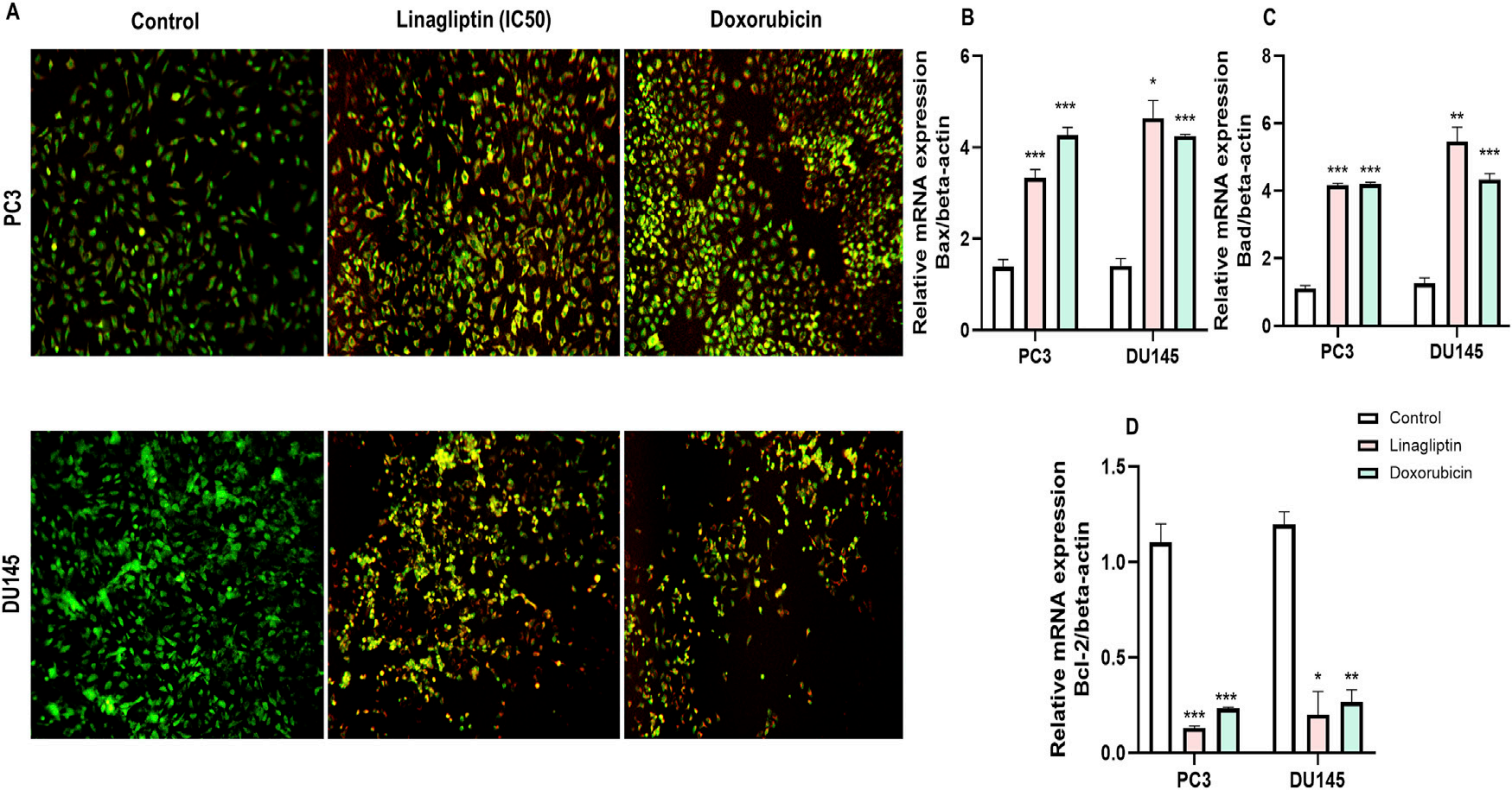
Linagliptin induces apoptosis in PC3 and DU145 prostate cancer cells. **(A)** AO/EtBr double staining assay–live cells and early apoptotic cells in green, with fragmented chromatin late apoptotic cells in orange and necrotic cells in bright red. **(B)** *Bax* expression **(C)** *Bad* expression **(D)** *Bcl-2* expression. The results are expressed in mean ± SD of three independent experiments. ***P < 0.001 vs. Control, **P < 0.01 vs. Control, *P < 0.05 Vs. Control.

**FIGURE 12:**
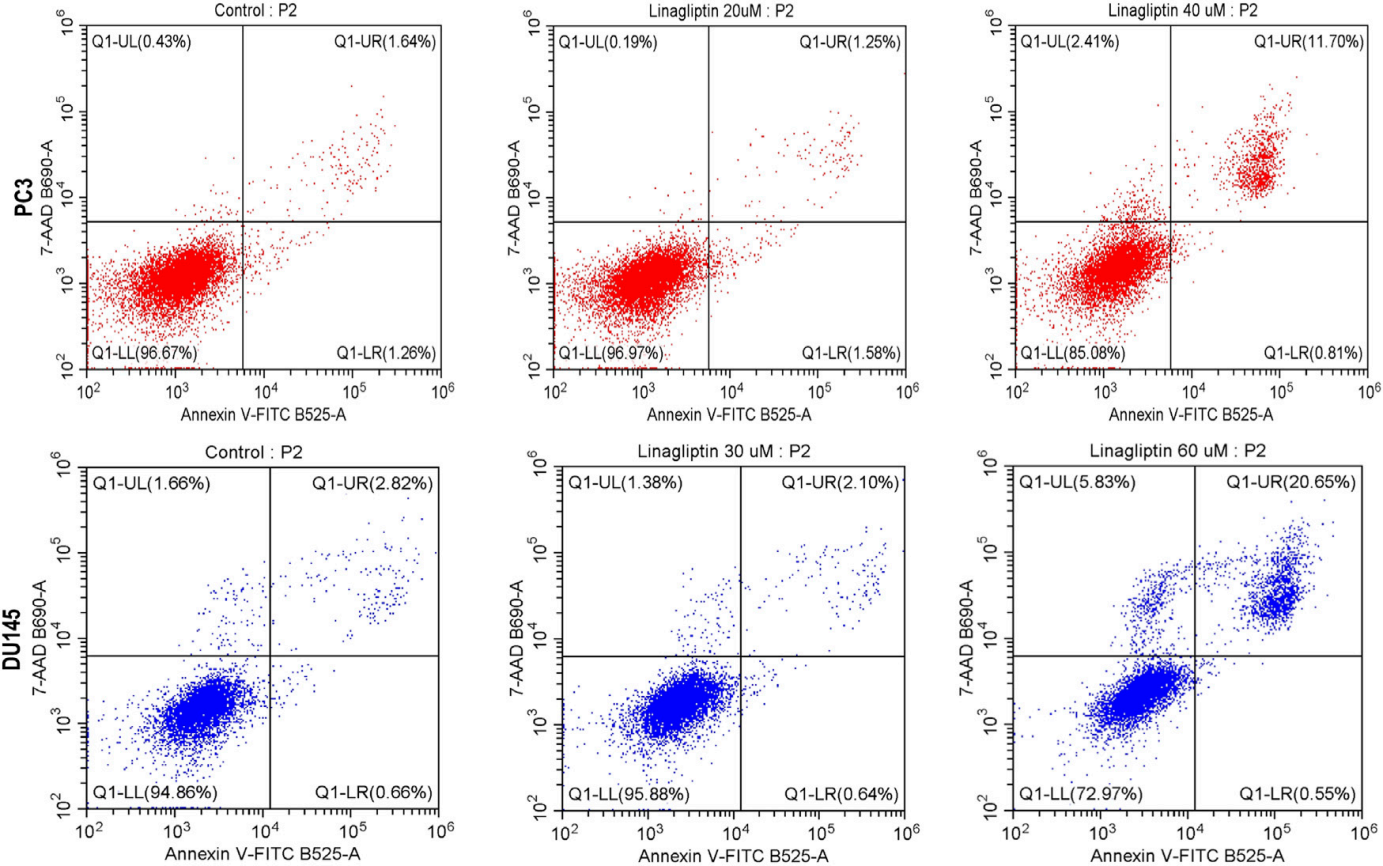
Apoptosis assay was performed with flow cytometry using PI and Annexin V-FITC double staining. Linagliptin induced apoptosis in PC3 and DU145 cells in a concentration-dependent manner.

To visually confirm cellular death induced by Linagliptin in PC3 and DU145 cells, we performed AO/EtBr dual staining. The principle behind dual staining is to distinguish between different stages of apoptosis. AO stains live cells green; however, loss in the cellular membrane integrity leads EtBr to enter and stain cells as red. Moreover, the stages can be divided into live cells in green color, early apoptotic cells in green with fragmented chromatin, late apoptotic cells in orange, and necrotic cells in bright red (Liu et al., 2015). As shown in Figure 11A, Linagliptin treatment has increased the number of early and late apoptotic cells in the field compared to non-treated control cells. On the other hand, the positive control drug doxorubicin produced more necrotic cells than Linagliptin-treated cells, suggesting the probability of inducing future inflammation. To further confirm the initiation of apoptosis, we checked the expression level of pro and anti-apoptotic proteins. Linagliptin significantly reduced the expression of anti-apoptotic *BCL-2* compared to the untreated control group and significantly increased the pro-apoptotic *Bax* and *Bad expression.* Linagliptin showed a better apoptotic induction than Doxorubicin (Figures 11B–D). In addition to microscopy and protein expression analysis, Annexin V-FITC/PI flow cytometry was performed to quantitatively assess apoptosis induction by Linagliptin. Flow cytometry data revealed a dose-dependent increase in apoptotic cell population, with the maximum apoptotic response up to 11.70% observed at 40 µM Linagliptin in PC3 cells, whereas 20.65% was observed in DU145 cells at 60 µM with early and late apoptotic cell populations significantly increased in both cell lines, confirming Linagliptin-induced apoptotic cell death (Figure 12).

#### 3.2.7 DPP4 inhibition downregulates FGF17 expression, and suppresses PI3K/AKT signaling

**FIGURE 13:**
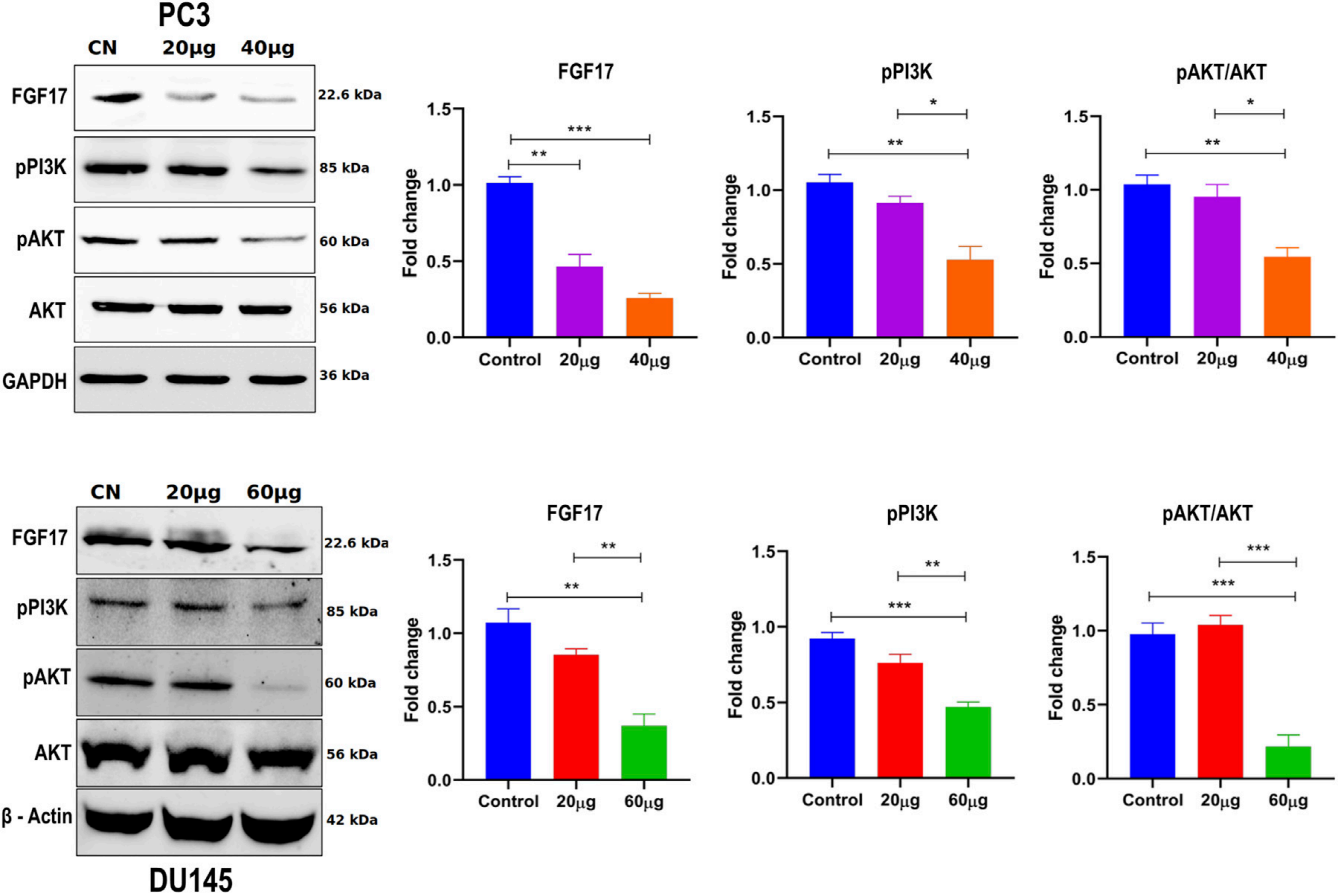
The effect of Linagliptin on *FGF17*, *PI3K/Akt* signaling pathway in PCa, A, B Expression of FGF17, PI3K/AKT in PCa, PC3 and DU145 both treated with varying concentrations of Linagliptin (0, 20, and 40 µM for PC3 and 0, 20, and 60 µM for DU145) for 24 h and cell lysates were subjected to western blot analysis with *FGF17*, PI3K, p-AKT and AKT anti-bodies, followed by sequential re-probing against GAPDH and beta-actin. The bar graph depicts densitometric expression analysis of FGF17, PTEN, PI3K, p-AKT and AKT. All the experiments were performed in triplicates and the data expressed as Mean ± SD. *P < 0.05, **P < 0.01, ***P < 0.001.

To validate the transcriptomic data, suppression of PI3K/Akt signaling upon DPP4 inhibition, we performed Western blot analysis in PC3 and DU145 cells treated with Linagliptin. DPP4 inhibition led to a marked reduction in the levels of phosphorylated PI3K (p-PI3K) and phosphorylated Akt (p-Akt), indicating suppression of the *PI3K/Akt* signaling axis (Figures 13A,B). Total Akt protein levels remained unchanged, suggesting that the observed decrease was due to reduced pathway activation rather than total protein depletion. GAPDH and β-actin were used as loading controls and showed consistent expression across all samples. These results confirm that DPP4 inhibition attenuates *PI3K/Akt* signaling at the protein level, supporting a mechanistic link between DPP4 activity and oncogenic pathway activation in PCa (Figure 13).

**FIGURE 14:**
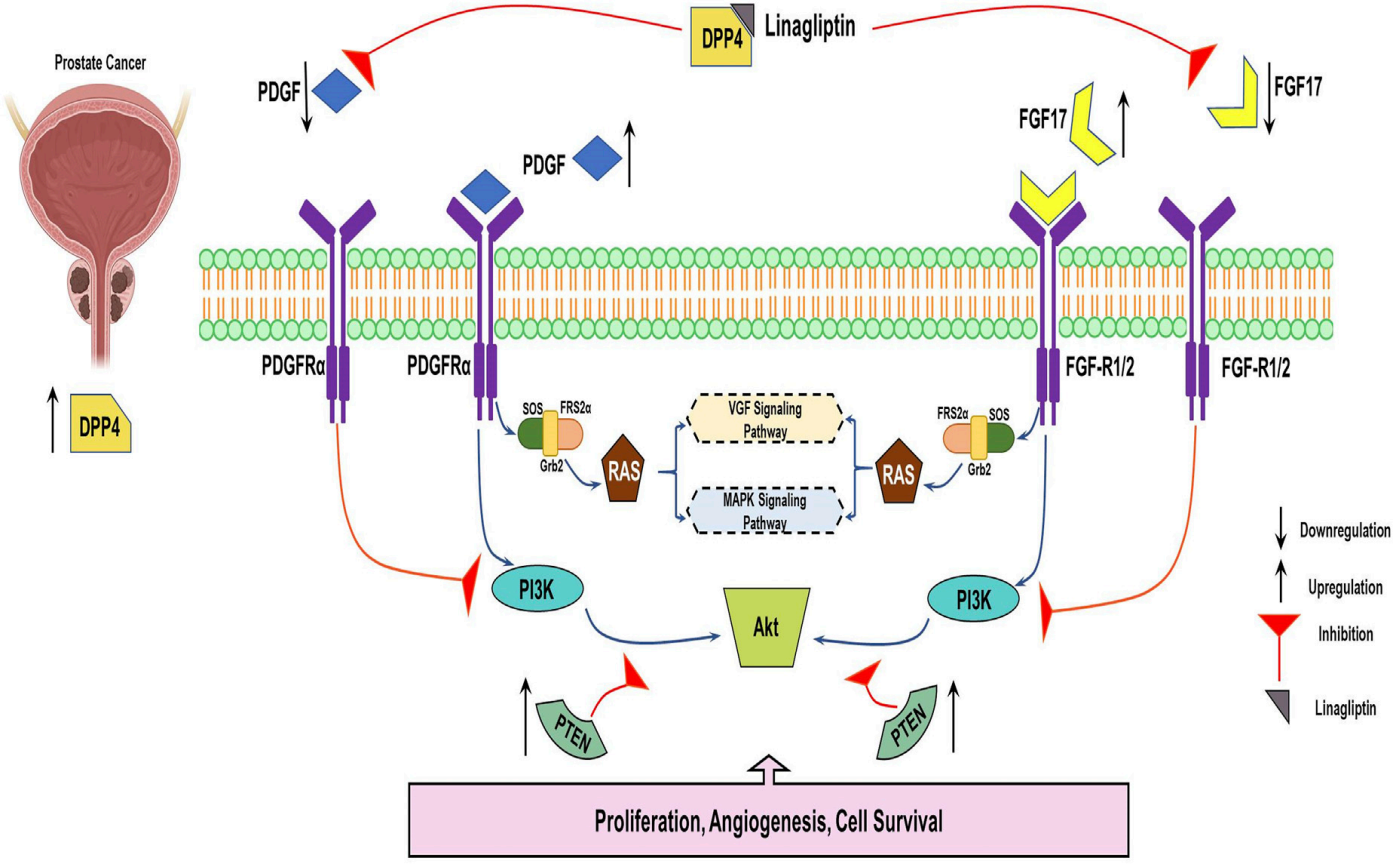
Systemic representation of DPP4 inhibitor downregulating FGF17and PDGF Prostate cancer progression via PI3K/AKT pathway.

This study clearly demonstrates that Linagliptin, a well-known DPP-4 inhibitor, can regulate the progression of prostate cancer cells by modulating the *PI3k/Akt* pathway and its key genes, as illustrated in the schematic presentation (Figure 14). Consequently, it induces apoptosis in prostate cancer cells *in vitro*. This highlights the *in vitro* molecular mechanism underlying Linagliptin’s anti-proliferative efficacy in prostate cancer.

## 4 Discussion

Prostate cancer (PCa) remains a significant clinical challenge due to its complex molecular heterogeneity and variable treatment responses. Our integrated genomic-transcriptomic investigation has systematically identified DPP4 as a critical molecular regulator of PCa progression through modulation of the *PI3K/Akt* signaling pathway. These findings provide novel mechanistic insights that advance our understanding of PCa pathogenesis and potential therapeutic interventions. The central role of *PI3K/Akt* signaling in PCa development and progression has been extensively documented (Wise et al., 2017). Our study builds upon this foundation by employing a comprehensive systems biology approach to identify 22 hub genes (Figures 2A–D) of which 8 genes (*FGF17, NGFR, NTRK1, KIT, PDGFRA, ITGA5, COL4A2, COL9A1*) significantly enriched in this pathway (Figures 3A–D). The frequent loss of *PTEN*, a key negative regulator of *PI3K/Akt* signaling observed in 40%–60% of advanced PCa cases (Jamaspishvili et al., 2018), Cross talk pathway analysis for those hub genes further confirms their role in the Androgen receptor (AR) and Mitogen-Activated Protein Kinase (MAPK) signaling pathways Supplementary Figure S2. Previous studies have reported DPP4’s involvement in AR signaling and MAPK pathways. For example, DPP4 has been shown to influence AR signaling and contribute to progression of castration-resistant PCa (Russo et al., 2018), as well as modulate MAPK-related FGF signaling (Wesley et al., 2005). According to Hashemi et al., 2023, *PI3k/Akt* pathway is frequently activated in advanced prostate cancer. Further DPP4 and *PI3k/Akt* pathway in prostate cancer is an area of ongoing research.

TCGA-PRAD cohort analysis revealed a strong association between elevated DPP4 expression and poor clinical outcomes was observed through overall survival analysis (Figures 4A–D), a finding that aligns with previous reports of DPP4 overexpression in metastatic PCa (Lu et al., 2022). However, our study significantly extends these observations by demonstrating that this prognostic association may be mechanistically linked to DPP4-mediated activation of *PI3K/Akt* signaling (Figures 5A–C). This connection provides a plausible explanation for the aggressive phenotype observed in DPP4-high tumors and suggests potential therapeutic vulnerabilities that could be exploited in clinical settings.

Treatment with the DPP4 inhibitor Linagliptin resulted in marked suppression of *PI3K/Akt* signaling pathway. The observed dose-dependent effects included: (i) significant downregulation of PI3K and AKT expression (Figure 7Aii associated upregulation of *PTEN*, (Figure 7Aiii) induction of G1/S cell cycle arrest (Figures 7B,C). This result strongly supports DPP4’s role as a key upstream regulator of this oncogenic signaling cascade in PCa.

Linagliptin’s anti-metastatic properties demonstrated superior efficacy compared to doxorubicin in both wound healing (Figures 8A,B) and transwell migration assays (Figures 9A,B), suggesting that DPP4 inhibition may represent a promising strategy for preventing or treating metastatic PCa. The molecular basis for this activity appears to involve DPP4’s regulation of extracellular matrix components and growth factor signaling pathways, particularly through modulation of *FGF17* and *PDGFRA* expression. The downregulation of *FGF17* and *PDGFRA* (Figures 10A, 13A,B) following DPP4 inhibition provides important context to previous studies linking these growth factors to PCa progression (Teishima et al., 2019). From Figure 10A, DPP4 inhibition also affects collagen genes (*COL4A1, COL9A2*) offers new insights into potential mechanisms of tumor microenvironment remodeling in PCa, a process that has been increasingly recognized as critical for cancer progression (Zhang et al., 2023). The shift in apoptotic balance we observed (decreased *BCL2* with increased *BAX* expression Figures 11B,C) following DPP4 inhibition further supports the growing recognition of DPP4’s role in cell survival pathways.

Our results strongly support the oncogenic function in primary PCa through *PI3K/Akt* activation, while some studies have suggested that the DPP4 role is influenced by tissue type, disease stage, and treatment context. DPP4 is found at higher levels in malignant prostate tissue compared to benign or normal prostate tissue. This increased expression is associated with PCa progression and correlates with factors such as prostate-specific antigen (PSA) levels, tumor size, and overall stage of the disease. The reasons behind these varying levels may be connected to the tumor microenvironment and how the tumor interacts with different growth factors and signaling pathways.

In the localized stage of prostate cancer, DPP4 activity may be relatively high, while in advanced metastatic disease, its activity can be reduced. This reduction in activity in advanced disease may be due to a low-molecular-weight inhibitor, suggesting a potential difference in the role of DPP4 in different stages of the disease. Large population-based studies indicate that DPP4 inhibitors confer a survival advantage across various disease stages.

Particularly after treatments like androgen deprivation therapy, this emphasizes the need for a nuanced understanding of its biological context in different phases of PCa. Various treatments, including chemotherapy, androgen deprivation therapy, prostatectomy, and radiation therapy, suggesting that the benefit is not limited to a specific subgroup. DPP4 inhibitors, commonly used for type 2 diabetes, have shown potential in treating advanced prostate cancer in some contexts by improving overall survival. However, the enzyme’s role can shift depending on the progression of the disease and the treatment. Furthermore, studies suggest that DPP4 may act as a tumor suppressor gene in the AR pathway, and its inhibition could potentially accelerate prostate cancer progression, particularly after androgen deprivation therapy.

These findings highlight the importance of considering tissue-specific expression, disease stage and treatment context when evaluating DPP4-targeted therapies in prostate cancer, as their efficacy appears closely tied to the unique biology of this tumor type (Russo et al., 2018; Shah et al., 2019; Mangoura et al., 2024). Such context-dependent behavior underscores the importance of careful patient selection in potential clinical applications of DPP4-targeted therapies. While Linagliptin was chosen for its relevance and specificity to DPP4, though we recognize the limitations of using a single agent and the possibility of off-target effects. Future studies will employ complementary methods, like DPP4-specific siRNA knockdown, and other DPP4 inhibitors, to better confirm DPP4’s role in *PI3K/Akt* signaling and ensure the observed effects are due to DPP4 inhibition. The clinical implications of our findings are particularly significant given that DPP4 inhibitors are already FDA-approved for diabetes management. Our results provide a strong mechanistic rationale for re-purposing these agents in PCa treatment, building upon epidemiological studies suggesting improved outcomes in diabetic PCa patients taking DPP4 inhibitors (Pan et al., 2021). The multi-modal effects suggest that DPP4 inhibitors could offer comprehensive therapeutic benefits in PCa management.

## 5 Conclusion

To understand the PCa heterogeneity, we integrated whole genome SNV data with transcriptomic profiles from TCGA-PRAD. Although the majority of SNVs were located in non-coding regions, a subset of exonic mutations, primarily missense variants were functionally annotated, revealing 3.806 genes with deleterious effect on protein function. Integration of these deleterious mutation genes with differentially expressed genes identified 357 common genes, including 22 hub genes, with significant enrichment in *PI3K/Akt* pathway. Among these hubs, DPP4 was found to modulate key regulators of this pathway, such as *FGF17, PDGFRA, COL4A1* and *COL9A2*. Our findings suggest that DPP4 inhibition via (Linagliptin) may suppress regulate *PI3K/Akt* signaling thereby impacting the cell survival and potentially suppressing PCa metastasis. These results highlight DPP4 as a promising therapeutic target for PCa, warranting further *in-vivo* validation and mechanistic studies.

## Data Availability

The original contributions presented in the study are included in the article/Supplementary Material, further inquiries can be directed to the corresponding author.
