# Supplementary material for: Integrative genomic analysis identifies DPP4 inhibition as a modulator of FGF17 and PDGFRA downregulation and PI3K/Akt pathway suppression leading to apoptosis
Source: Front Pharmacol. 2025 Jul 22;16:1606914. doi: 10.3389/fphar.2025.1606914 (PMC12321844; doi:10.3389/fphar.2025.1606914)
Supplement: Supplementary file 1 [file Supplementaryfile1.docx]

| RTN1 | PALLD | LHFPL2 | KCNN2 | GTF2IRD2B | CNN2 | DSC2 | NEXN |
| --- | --- | --- | --- | --- | --- | --- | --- |
| ZEB1 | CPLX3 | RHBDF1 | MAPK8IP3 | SYNE1 | AKR1B1 | ABCC4 | PEX10 |
| HIGD1B | SERPINB9 | ADCY3 | BOC | CANT1 | NSMCE1 | SLC43A3 | KLC1 |
| ROR2 | KNTC1 | WNT6 | SOBP | PRDM5 | ABCA9 | ATG16L2 | QSOX1 |
| RASA4B | EFHC1 | CLPS | CPT1C | PER1 | TGFBR3 | ARHGEF40 | BMPR1B |
| TF | NACAD | LAMA3 | LPIN1 | DCHS1 | ATP6V0E2 | SDK1 | CYP2J2 |
| TTLL4 | SRD5A2 | SPEG | ITIH4 | TWIST1 | COL27A1 | CAV1 | SLC39A6 |
| DNHD1 | MEIS1 | IGHMBP2 | SLC17A5 | ST6GALNAC1 | SRRM2 | DHDH |  |
| ZNF350 | CACNA1H | HIC1 | CYBA | CHRDL1 | C1QTNF1 | ARHGEF17 | |
| FHL1 | LRBA | STAB1 | STX2 | LAMA2 | IQGAP2 | ABCA7 |  |
| LPIN3 | KRT8 | TMC5 | DPP4 | NID1 | MICU3 | GMDS |  |
| PPARGC1A | DENND2A | TCERG1 | GPR173 | BCO2 | SEC23B | RASGRF2 |  |
| CENPJ | DMTF1 | PDGFRA | EVC2 | PAN2 | CPLX1 | MBOAT2 |  |
| EHBP1L1 | BNIPL | PGM5 | SEC31B | FADS2 | KCNH2 | AHDC1 |  |
| VWCE | MYOCD | ADD3 | ADAMTS9 | OTX1 | NRP2 | AATK |  |
| ANKDD1A | MIPEP | PKD1 | ARHGEF2 | TM7SF2 | FES | SPPL2B |  |
| RDH11 | CASQ1 | ANKRD10 | AVIL | TMEM132C | MYOF | ACSL3 |  |
| UCKL1 | COL5A3 | RBM33 | RNF112 | EDNRB | ANO1 | UTRN |  |
| LCLAT1 | COL4A2 | ITGA8 | RSPO1 | JAZF1 | PLXNA2 | RNF217 |  |
| SPTBN2 | NEK5 | MRC2 | DPY19L2 | SOAT1 | CYTL1 | ACSF2 |  |
| HBB | ANGPTL4 | MYO18A | NXF1 | TMEM100 | SORBS1 | WDR91 |  |
| PGR | AKAP12 | STAG3 | COL13A1 | TUBE1 | SLC18A2 | COL5A2 |  |
| DMD | ZNF423 | NLN | RIN1 | B3GNT8 | TTLL3 | MAP7 |  |
| SLIT2 | NTF3 | PABPC1L | STK11IP | COL4A4 | ZEB2 | GABRG3 |  |
| FAM3D | CLIP4 | CAPRIN2 | KLF8 | GRIK5 | CCDC146 | ARGLU1 |  |
| SLC4A4 | GABRB3 | TNFRSF10B | EPB41L2 | ATAD3C | CCND2 | SAMD5 |  |
| RHOBTB1 | DZIP1L | OXER1 | RGS9 | PSAT1 | ADAMTS10 | MEGF6 |  |
| DTNA | F5 | COL6A3 | CHRD | CCDC82 | HSPG2 | COL9A1 |  |
| COLQ | FAM222A | DHX35 | ADH1B | AEBP1 | ITGA5 | TYK2 |  |
| CLGN | MAST2 | RAB3IP | LRP4 | KIAA1614 | COL4A5 | SAMD11 |  |
| CLHC1 | S100P | MIB2 | TUBGCP6 | RARG | PHYHIP | TRPV4 |  |
| DSP | SOX13 | ALCAM | P4HB | HAAO | RASIP1 | SRD5A3 |  |
| MINK1 | HIF3A | POGZ | ACTA2 | TNFAIP2 | IDS | FUCA1 |  |
| ATP2C1 | FOLH1 | ZZEF1 | PMEPA1 | HSD11B1 | JPH2 | ZNF169 |  |
| PLA1A | MYBL2 | RASSF5 | DNAH5 | AHNAK2 | SC5D | IGSF9B |  |
| SPON1 | IP6K3 | POU6F1 | TP63 | PTH1R | CYP4F12 | PTPRU |  |
| INPP1 | SMIM10 | CPAMD8 | MMP9 | C1orf116 | SLIT1 | HSPA12A |  |
| NFKBID | NYNRIN | ZNF711 | SAMD4A | FBLN5 | NR4A1 | UCHL1 |  |
| PPP1R12C | HR | NCAPD3 | NINL | CNTNAP1 | RBM5 | GGA3 |  |
| C7 | SPATA6 | KIT | GRK5 | CSF3R | SCN7A | RAP1GAP |  |
| RNF165 | PARP6 | CAND2 | PCCB | PDIA3 | MAPKBP1 | SRPK3 |  |
| MYO15A | CPA6 | ADRA1D | SMTNL2 | FGF17 | FBXO2 | ARL10 |  |
| SIL1 | SLCO4A1 | TMEFF2 | NADSYN1 | WDR59 | BTAF1 | REV3L |  |
| CLEC1A | SCARA5 | COLEC12 | AQP11 | ATOH8 | ARHGEF10L | ADAM10 |  |
| DDR2 | SGCA | FGD2 | EHD2 | PLA2G6 | STT3A | SPTBN5 |  |
| GATM | TCIRG1 | MYLK | MAP3K6 | TDRD10 | PLIN4 | LAMA5 |  |
| DAPL1 | DST | SFI1 | DGCR8 | SYTL4 | PTPRZ1 | TMPRSS2 |  |
| ZNF600 | KLHL29 | BMP1 | EXOC3L4 | NGFR | DGKH | OSR1 |  |
| PLCE1 | UPP1 | AKAP8L | FAM153B | PLXND1 | PLXNB1 | SPDEF |  |
| SLC38A5 | NTRK1 | GLI2 | GPR161 | GALNT15 | LIPE | COL14A1 |  |

**Supplementary Table. 1:** List of 357 common genes
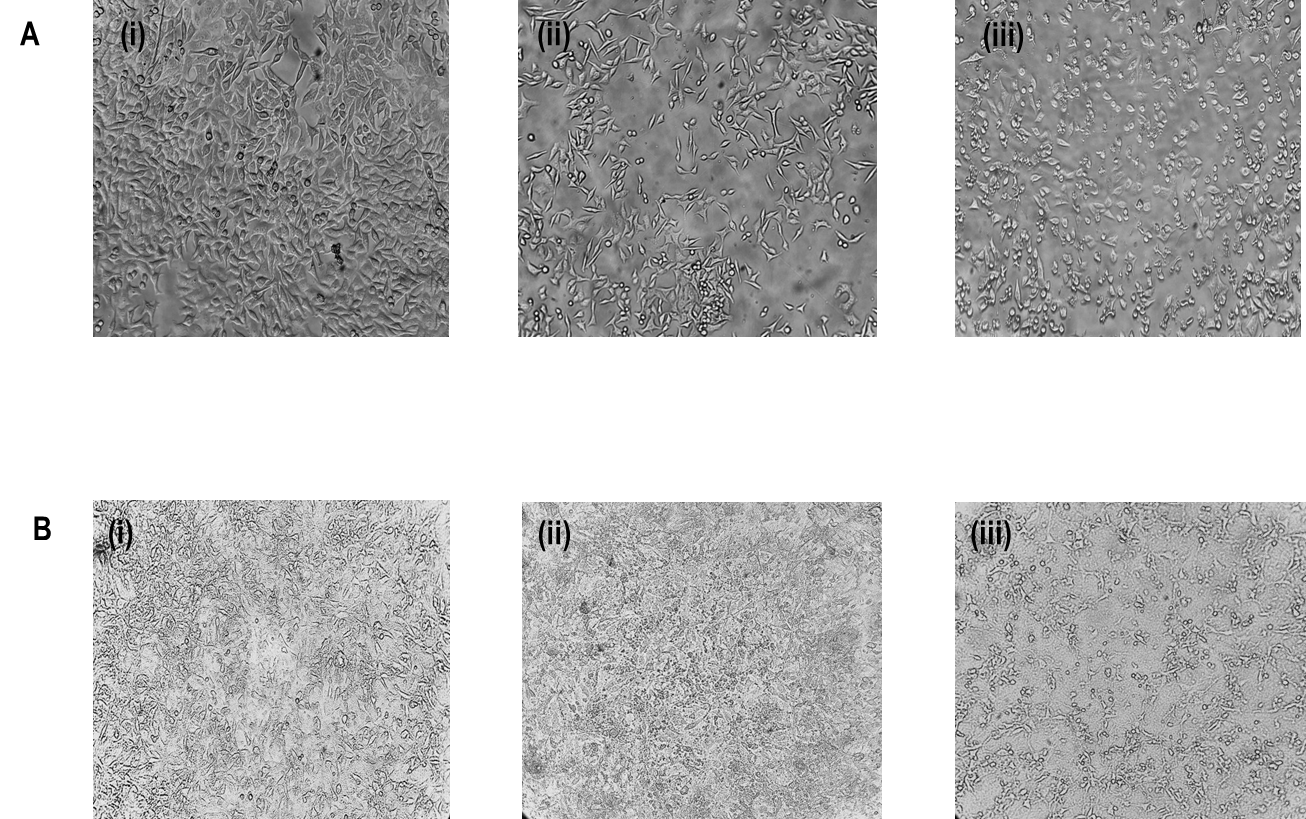


**Supplementary Fig. 1:** Linagliptin induces morphological changes in PC3, DU145 when treated with Linagliptin at 3µM. A(i) PC3 cells without treatment showing healthy morphology, (ii) PC3 cells treated with Linagliptin showing altered morphology, (iii) PC3 cells treated with Doxorubicin showing altered morphology. B (i) DU145 cells without treatment showing healthy morphology, (ii) DU145 cells showing altered morphology, (iii) DU145 cells treated with Doxorubicin showing altered morphology.


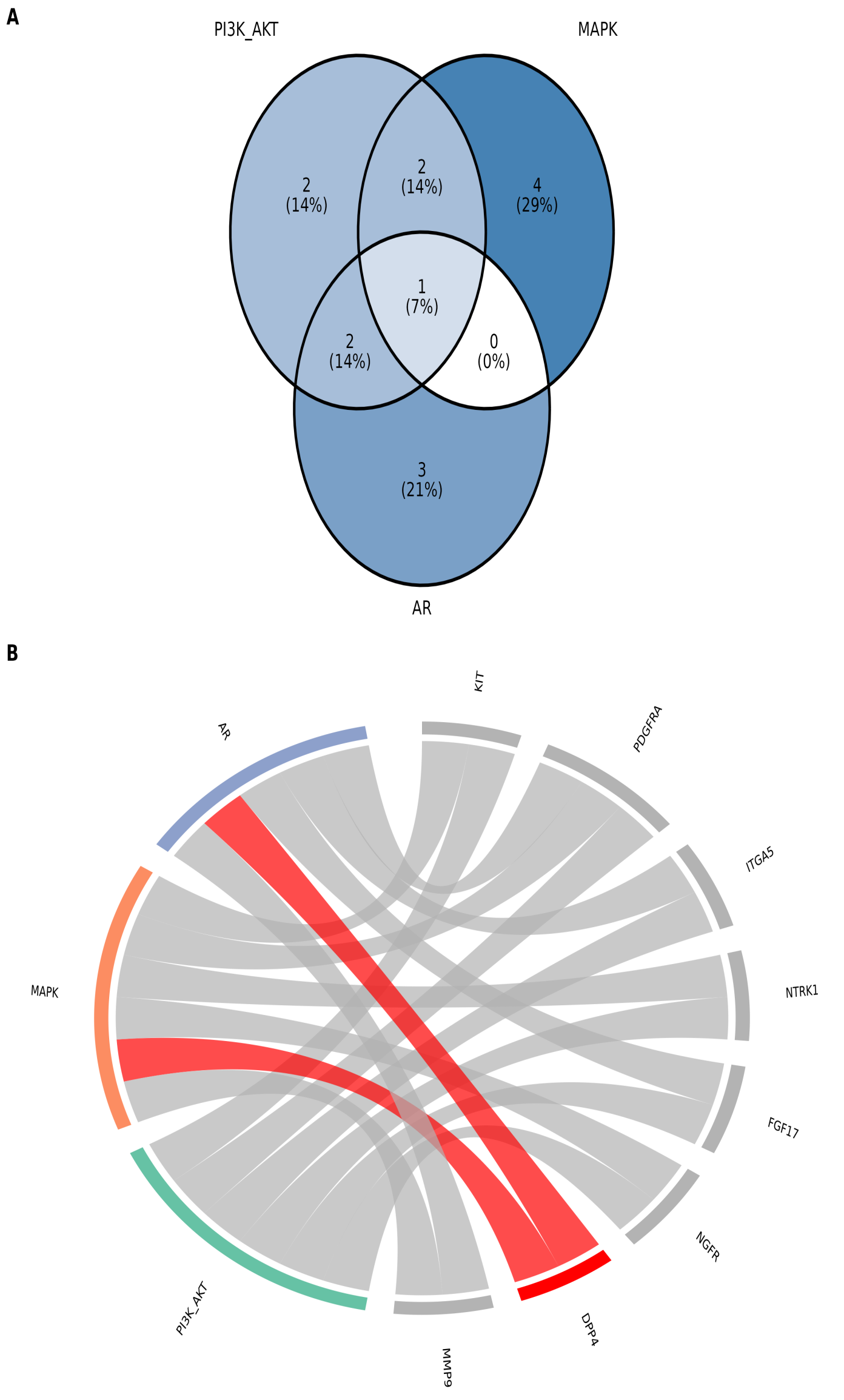


**Supplementary Fig. 2:** Interaction of hub genes in the signaling pathways

| node_name | MCC | Degree | Closeness | Betweenness |
| --- | --- | --- | --- | --- |
| 9606.ENSP00000348385 | 3628806 | 11 | 25.5 | 3.86667 |
| 9606.ENSP00000397908 | 14 | 3 | 16.58333 | 4.58333 |
| 9606.ENSP00000470152 | 6 | 12 | 27.66667 | 16.56995 |
| 9606.ENSP00000324532 | 3314 | 15 | 29 | 12.89162 |
| 9606.ENSP00000379866 | 3639840 | 2 | 15.41667 | 0 |
| 9606.ENSP00000302177 | 2 | 4 | 21.33333 | 157.4374 |
| 9606.ENSP00000350012 | 8 | 15 | 29.5 | 45.82374 |
| 9606.ENSP00000244289 | 13 | 9 | 27.33333 | 81.80241 |
| 9606.ENSP00000400365 | 2246 | 11 | 27.16667 | 6.59939 |
| 9606.ENSP00000354923 | 67 | 7 | 24.75 | 17.96756 |
| 9606.ENSP00000252999 | 6048 | 2 | 19.66667 | 0 |
| 9606.ENSP00000354487 | 18 | 5 | 23.5 | 5.2602 |
| 9606.ENSP00000325120 | 10 | 2 | 18.91667 | 1.33333 |
| 9606.ENSP00000264187 | 6546 | 16 | 30.83333 | 66.39101 |
| 9606.ENSP00000300289 | 9 | 5 | 25.5 | 13.72513 |
| 9606.ENSP00000305988 | 10 | 5 | 24 | 5.86688 |
| 9606.ENSP00000351338 | 2 | 13 | 28.16667 | 26.90212 |
| 9606.ENSP00000376303 | 12 | 2 | 20.58333 | 0 |
| 9606.ENSP00000327801 | 3633961 | 2 | 19.66667 | 0 |
| 9606.ENSP00000334198 | 1 | 7 | 25.16667 | 26.51908 |
| 9606.ENSP00000431418 | 19 | 12 | 27.16667 | 5.28136 |
| 9606.ENSP00000349790 | 3635280 | 16 | 29.5 | 16.04338 |
| 9606.ENSP00000331902 | 3641304 | 14 | 31.5 | 507.2804 |
| 9606.ENSP00000339191 | 29 | 11 | 28.41667 | 74.95742 |
| 9606.ENSP00000288135 | 80 | 6 | 24.83333 | 9.14283 |
| 9606.ENSP00000305714 | 12 | 3 | 20.08333 | 48.83413 |
| 9606.ENSP00000264867 | 7 | 11 | 28.91667 | 71.82722 |
| 9606.ENSP00000224784 | 446 | 8 | 25.83333 | 79.19976 |
| 9606.ENSP00000449404 | 11 | 6 | 24.66667 | 6.17224 |
| 9606.ENSP00000356515 | 36 | 6 | 24.25 | 23.01978 |
| 9606.ENSP00000487174 | 12 | 15 | 31.33333 | 181.8072 |
| 9606.ENSP00000257290 | 257 | 22 | 35.16667 | 415.3958 |
| 9606.ENSP00000361405 | 738 | 17 | 31.33333 | 47.93399 |
| 9606.ENSP00000297848 | 3631398 | 6 | 24.33333 | 11.43488 |
| 9606.ENSP00000172229 | 40 | 11 | 28.83333 | 172.0055 |
| 9606.ENSP00000242261 | 61 | 15 | 30.16667 | 65.45725 |
| 9606.ENSP00000363827 | 3106 | 3 | 21.83333 | 7.00153 |
| 9606.ENSP00000367316 | 3360 | 12 | 28 | 13.95449 |
| 9606.ENSP00000352414 | 74 | 9 | 27.25 | 32.1623 |
| 9606.ENSP00000345008 | 34 | 20 | 33 | 85.19068 |
| 9606.ENSP00000295550 | 3640968 | 5 | 22.08333 | 19.23027 |
| 9606.ENSP00000364000 | 3636302 | 17 | 31.33333 | 53.2657 |
| 9606.ENSP00000293379 | 12875 | 21 | 34.5 | 317.3775 |
| 9606.ENSP00000262018 | 24 | 5 | 23.33333 | 2.52617 |
| 9606.ENSP00000264828 | 3628926 | 12 | 26.33333 | 6.65372 |
| 9606.ENSP00000397297 | 30 | 11 | 25.83333 | 2.78271 |
| 9606.ENSP00000381949 | 3628824 | 5 | 25.66667 | 58.6138 |
| 9606.ENSP00000353731 | 8 | 2 | 21.33333 | 3.29073 |
| 9606.ENSP00000353654 | 3644046 | 20 | 33.5 | 153.4043 |
| 9606.ENSP00000353582 | 4 | 3 | 22.58333 | 3.2132 |

**Supplementary Table. 2:** Topological Score of top Maximal Clique Centrality (MCC), Degree Centrality, Closeness Centrality, Betweenness Centrality.

| 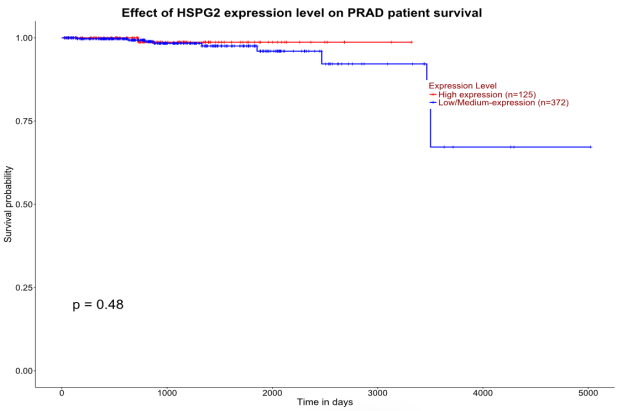 | 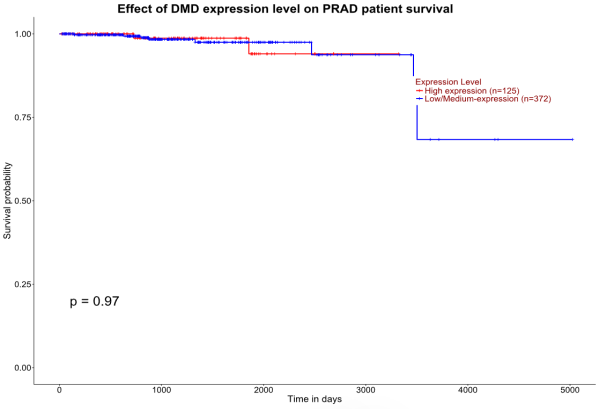 | 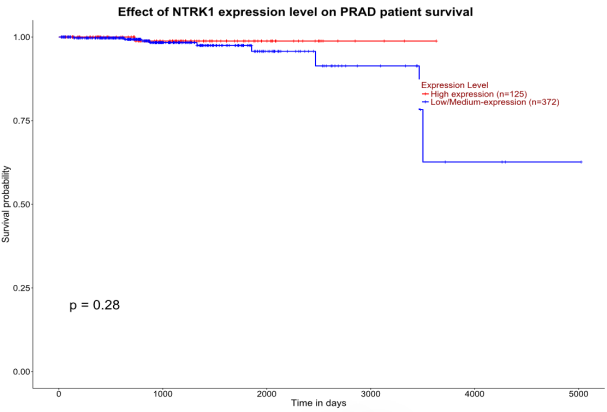 |
| --- | --- | --- |
| 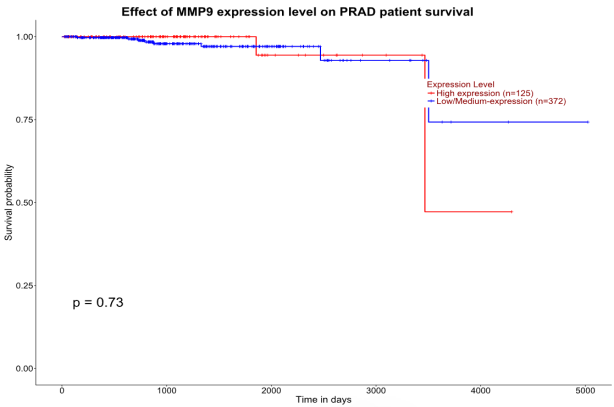 | 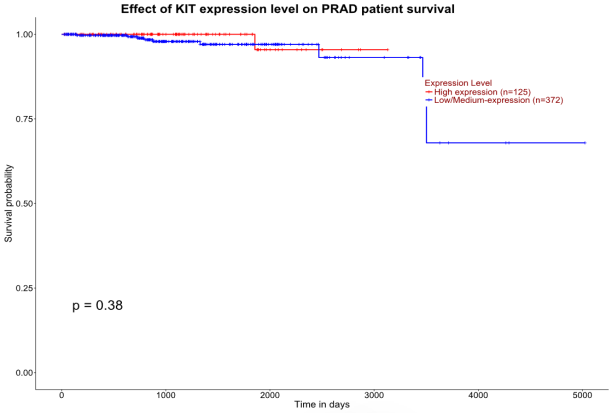 | 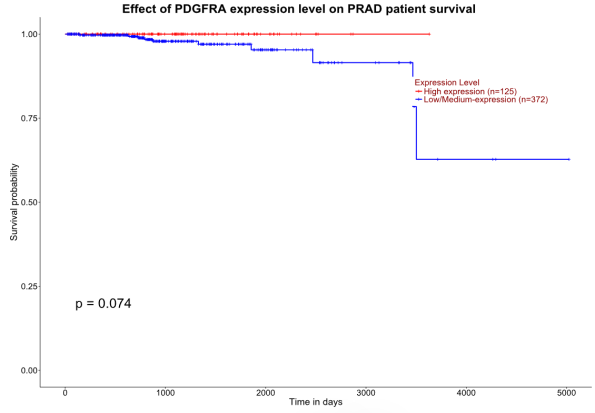 |
| 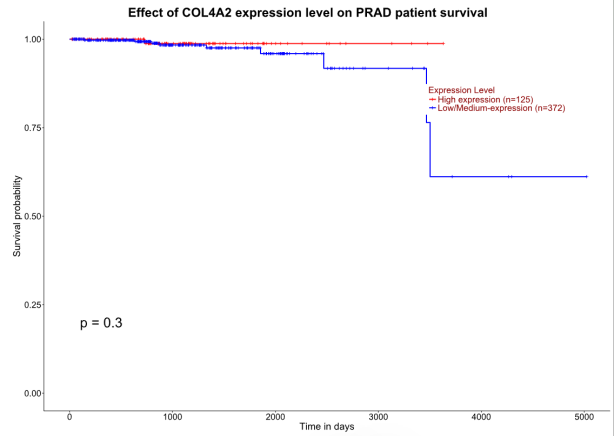 | 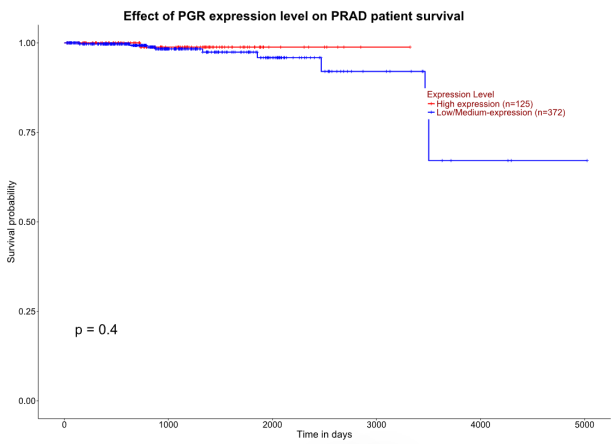 | 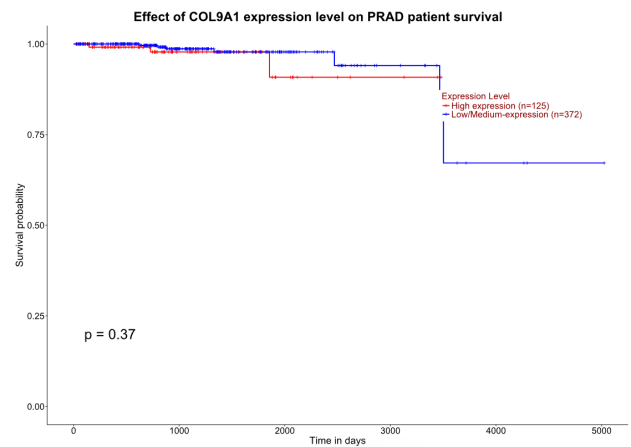 |

**Supplementary Fig. 3.** Kaplan-Meier (KM) overall survival analysis of top 10 (excluded DPP4) using UALCAN database TCGA-PRAD cohort (log-rank p-value < 0.001)
